# Supplementary material for: Pharmacology, Pharmacotherapy, and Pharmacopolicy Through an Evidence-Based Medicine: A Novel Approach for First-Year Medical Students
Source: MedEdPORTAL. 2020 Jul 20;16:10934. doi: 10.15766/mep_2374-8265.10934 (PMC7373350; doi:10.15766/mep_2374-8265.10934)
Supplement: Supplementary file 1 — Activity Information.docxUSDA QuickSheet.pdfFDA QuickSheet.pdfAdverse vs Side Effects.docxSeating Chart.pdfAcetaminophen Handout.pdfBeano Handout.docxMevacor Handout.pdfNaproxen Handout.pdfPraluent Handout.pdfXenical Handout.pdfFat-Soluble Vitamins Handout.pdfGroup Quiz.docxQuiz Answers.docx [file mep_2374-8265.10934-s001.zip › A. Activity Information.docx]

**SIG (Small Interactive Group) 3- Drugs and Dietary Supplements**

**Regulatory Oversight by the Food and Drug Administration (FDA)**

Alexander Mozeika, PharmD*, Rijul Asri, B.S.**, James Theis, Ph.D. and Carolyn K. Suzuki, Ph.D.

* NJMS MD candidate, ** NJMS MD/PhD candidate

**Objectives:**

1. By the end of this exercise, first-year medical students will be able to apply the regulatory principles of the Food and Drug Administration (FDA) and United States Department of Agriculture (USDA) in the context of the evaluation of clinical trials and drug markets

2. By the end of this exercise, first-year medical students will be able to compare and contrast FDA-approved prescription and generic drugs, OTC drugs, and dietary supplements in the development of patient-specific care plans

3. By the end of this exercise, first-year medical students will be able to apply knowledge of mechanisms of action, intended health outcomes, and potential adverse effects of the specific drugs and dietary supplements assigned in the appendices to determine the best course of action for treatment and monitoring in clinical scenarios

4. By the end of this exercise, first-year medical students will be able to integrate theoretical knowledge with targeted, evidence-based consultation of drug databases in order to make real-time optimal treatment decisions for specific clinical scenarios

**Required preparation:**

1. Background Information (see below). Learn this material before coming to SIG 3.
2. “Overview- Drug Development Process”. Learn before coming to SIG 3.
3. “Overview- FDA Drug Approval Process”. Learn before coming to SIG 3.
4. Adverse Effects versus Side Effects. Learn before coming to SIG 3.
5. Familiarize yourself with Lexicomp Online, *which you could use for in-class learning.*

Lexicomp is an invaluable resource available through the Rutgers Library system for accessing information about prescription and OTC drugs, as well as compounds and chemicals (e.g. caffeine, biotin, iron). Information about doses, mechanisms of action, drug interactions and adverse effects is available, along with patient education materials and other clinical tools.

Optional exercise: search “cyclosporine”, which is used to suppress the immune system to:

prevent rejection of organ transplants

treat rheumatoid arthritis
treat psoriasis
treat dry eye

**Format of SIG3**

**Part 1. Focused study**

- Pairs of students from each SIG group will be assigned to learn about specific prescription drugs or OTC drugs/dietary supplements. There will be a ~30 min in-class self-study learning period.
- Each pair of students will receive a packet of material about their assigned drugs, which is color-coded. FIVE COLOR PAIRS will gather to study and discuss their topics and background material. For example, the orange pairs from SIG groups 1 through 5 will get together. Locations of color-code groups in the auditorium will be posted during SIG3.
- Your study material will include a list of questions or talking points relevant to the assigned drug/supplement. For example:

What is the intended benefit of the drug/supplement?

What are their molecular mechanisms of action?

What are potential side effects?

**Part 2. Teaching/discussion**

After the focused study period, SIG groups will re-assemble and each pair of students will teach the group about their set of drugs or dietary supplements. The teaching/discussion period will be 30-45 min.

**Part 3. Group Quiz**

After the teaching/discussion period, there will be a Group Quiz based on - 1) the assigned pre-SIG preparation material, and 2) the in-class activity.

**Background Information**

**Background information (**below), and the other **Required Preparation** material (additional pdf files), will provide information about the FDA and their regulatory activities relevant to human health and nutrition. Information is also provided about FDA control of supplements and the drug approval process.

**Definitions**

**United States Food and Drug Administration (USFDA or FDA)**

- Within the Department of Health and Human Services (HHS), which is an Executive Branch of the US government.
- Controls and supervises:
  - food safety
  - tobacco products
  - dietary supplements
  - prescription and over-the-counter medications
  - vaccines
  - biopharmaceuticals
  - blood transfusions
  - medical devices
  - electromagnetic radiation emitting devices (ERED)
  - cosmetics
  - animal foods & feed
  - veterinary products

As of 2017, 75% of the FDA budget (~$700 million) is funded by the pharmaceutical company application fees due to the Prescription Drug User Fee Act.

**United States Department of Agriculture** (**USDA)**- an Executive Branch of the US government responsible for developing and executing federal laws related to farming, agriculture, forestry, and food.

*An Advisory Committee of Scientists submits an Advisory Report to the Secretaries of HHS and USDA regarding Dietary/Nutritional Guidelines.*

**Over-the-Counter (OTC) Drugs**. Over-the-counter medicine is also known as OTC or nonprescription medicine. All these terms refer to medicine that you can buy without a prescription. They are safe and effective when you follow the directions on the label and as directed by your health care professional.

**Prescription drugs**. Pharmaceutical drugs that requires a legal medical prescription for dispensation. By contrast, OTC drugs do not require a prescription. Learn prep material about “Overview- Drug Development Process” and “FDA Drug Approval Process” (see pdf files).

**Generic Drug Approval Process**

Once the patent for a novel drug entity has expired (20 years from date of filing which occurs years before widespread marketing), companies are able to create a generic product. The official definition of a generic drug as decided by the FDA is a drug “that is comparable to an innovator drug product in dosage form, strength, route of administration, quality, performance characteristics and intended use”. Although a generic drug contains the identical chemical moiety in the same quantity that the innovator product contains, it may differ in the excipients (inactive ingredients) incorporated into the finished drug product. Since these generic products are essentially the same as the branded product, the FDA has waived the requirement for proving safety and efficacy. However, to make this assumption that safety and efficacy are not altered, the company must produce pharmacokinetic studies and demonstrate comparable plasma concentration versus time profiles, also referred to as bioequivalence. Lastly, after these studies are completed, the generic drug company files an Abbreviated New Drug Approval (ANDA) application and not the traditional NDA application. This process was established as a mechanism to help reduce the cost incurred by a generic drug company during the drug development process, so ultimately these drugs can be made available at a lower cost to patients.


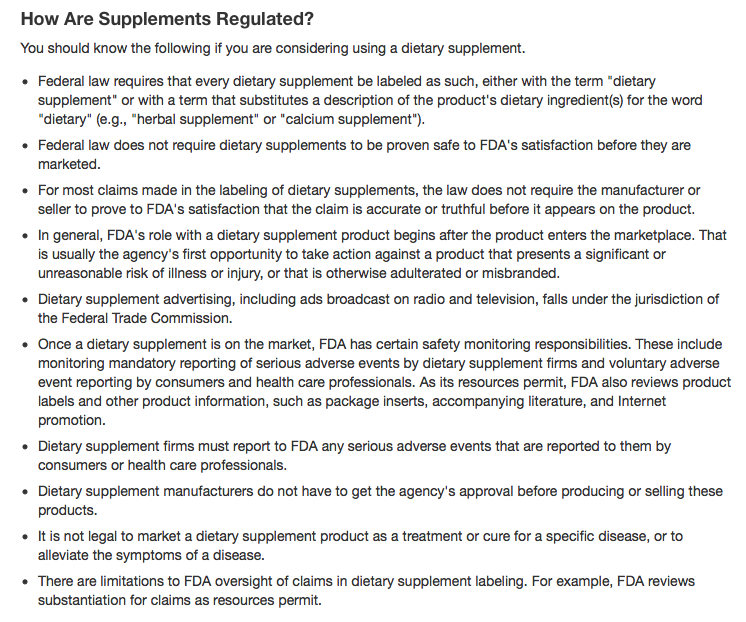


**WARNINGS AND PRECAUTIONS**

FDA regulated labeling requirements for prescription drugs, describing contraindications, boxed warning sections of labeling, and determination of which adverse reactions or other potential safety hazards are significant enough to warrant inclusion in the WARNINGS AND PRECAUTIONS section.

**CONTRAINDICATIONS**

A contraindication is a specific situation in which a drug, procedure, or surgery should not be used because it may be harmful to the person. There are two types of contraindications:

**Relative contraindication** means that caution should be used when two drugs or procedures are used together. However, it is acceptable to do so if the benefits outweigh the risk.

**Absolute contraindication** means that an event or substance could cause a life-threatening situation. A procedure or medicine that falls under this category must be avoided. Some treatments may cause unwanted or dangerous reactions in people with allergies, high blood pressure, or pregnancy. For example, isotretinoin, a drug used to treat acne is absolutely contraindicated in pregnancy due to the risk of birth defects. Certain decongestants are contraindicated in people with high blood pressure and should be avoided. Many medicines should not be used together by the same person. For instance, a person who takes warfarin to thin the blood should not take aspirin, which is a blood thinner. This is an example of a relative contraindication.

**ADVERSE REACTIONS**

Also see required pdf describing the difference between adverse reactions and side effects.

In pharmacology, adverse reactions are any unexpected or dangerous reaction to a drug. An unwanted effect caused by the administration of a drug. The onset of the adverse reaction may be sudden or develop over time.

**Dose-related adverse drug reactions** represent an exaggeration of the drug's therapeutic effects. For example, a person taking a drug to reduce high blood pressure may feel dizzy or light-headed if the drug reduces blood pressure too much. A person with diabetes may develop weakness, sweating, nausea, and palpitations if insulin or an oral antidiabetic drug reduces the blood sugar level too much. This type of adverse drug reaction is usually predictable but sometimes unavoidable. It may occur if a drug dose is too high [(overdose reaction)](https://www.merckmanuals.com/home/drugs/adverse-drug-reactions/overdose-toxicity), if the person is unusually sensitive to the drug, or if another drug slows the metabolism of the first drug and thus increases its level in the blood. Dose-related reactions are usually not serious but are relatively common.

**Allergic drug reactions** are not dose-related but require prior exposure to a drug. [Allergic reactions](https://www.merckmanuals.com/home/immune-disorders/allergic-reactions-and-other-hypersensitivity-disorders/overview-of-allergic-reactions) develop when the body's immune system develops an inappropriate reaction to a drug (sometimes referred to as sensitization). After a person is sensitized, later exposures to the drug produce one of several different types of allergic reaction. Sometimes doctors do [skin tests](https://www.merckmanuals.com/home/immune-disorders/allergic-reactions-and-other-hypersensitivity-disorders/overview-of-allergic-reactions#v27305662) to help predict allergic drug reactions.

**Idiosyncratic adverse drug reactions** result from mechanisms that are not currently understood. This type of adverse drug reaction is largely unpredictable. Examples of such adverse drug reactions include rashes, jaundice, anemia, a decrease in the white blood cell count, kidney damage, and nerve injury that may impair vision or hearing. These reactions tend to be more serious but typically occur in a very small number of people. Affected people may have genetic differences in the way their body metabolizes or responds to drugs.

Some adverse drug reactions are not related to the drug's therapeutic effect but are usually predictable, because the mechanisms involved are largely understood. For example, stomach irritation and bleeding often occur in people who regularly use aspirin or other [nonsteroidal anti-inflammatory drugs (NSAIDs)](https://www.merckmanuals.com/home/brain,-spinal-cord,-and-nerve-disorders/pain/treatment-of-pain#v734695). The reason is that these drugs reduce the production of prostaglandins, which help protect the digestive tract from stomach acid.
