# Supplementary material for: Pharmacology, Pharmacotherapy, and Pharmacopolicy Through an Evidence-Based Medicine: A Novel Approach for First-Year Medical Students
Source: MedEdPORTAL. 2020 Jul 20;16:10934. doi: 10.15766/mep_2374-8265.10934 (PMC7373350; doi:10.15766/mep_2374-8265.10934)
Supplement: Supplementary file 1 — Activity Information.docxUSDA QuickSheet.pdfFDA QuickSheet.pdfAdverse vs Side Effects.docxSeating Chart.pdfAcetaminophen Handout.pdfBeano Handout.docxMevacor Handout.pdfNaproxen Handout.pdfPraluent Handout.pdfXenical Handout.pdfFat-Soluble Vitamins Handout.pdfGroup Quiz.docxQuiz Answers.docx [file mep_2374-8265.10934-s001.zip › B. USDA QuickSheet.pdf]

Development & Approval Process (Drugs)

- **Developing New Drugs**
- **FDA Approval: What it means**

Developing New Drugs

American consumers benefit from having access to the safest and most advanced pharmaceutical system in the world. The main consumer watchdog in this system is FDA's Center for Drug Evaluation and Research (CDER).

The center's best-known job is to evaluate new drugs before they can be sold. CDER's evaluation not only prevents quackery, but also provides doctors and patients the information they need to use medicines wisely. The center ensures that drugs, both brand-name and generic, work correctly and that their health benefits outweigh their known risks.

Drug companies seeking to sell a drug in the United States must first test it. The company then sends CDER the evidence from these tests to prove the drug is safe and effective for its intended use. A team of CDER physicians, statisticians, chemists, pharmacologists, and other scientists reviews the company's data and proposed labeling. If this independent and unbiased review establishes that a drug's health benefits outweigh its known risks, the drug is approved for sale. The center doesn't actually test drugs itself, although it does conduct limited research in the areas of drug quality, safety, and effectiveness standards.

Before a drug can be tested in people, the drug company or sponsor performs laboratory and animal tests to discover how the drug works and whether it's likely to be safe and work well in humans. Next, a series of tests in people is begun to determine whether the drug is safe when used to treat a disease and whether it provides a real health benefit.

For more information about the drug development and approval process, see **How Drugs Are Developed and Approved (/Drugs/DevelopmentApprovalProcess/HowDrugsareDevelopedandApproved/default.htm)**.

FDA Approval: What it means

FDA approval of a drug means that data on the drug’s effects have been reviewed by CDER, and the drug is determined to provide benefits that outweigh its known and potential risks for the intended population. The drug approval process takes place within a structured framework that includes:

- *Analysis of the target condition and available treatments*—FDA reviewers analyze the condition or illness for which the drug is intended and evaluate the current treatment landscape, which provide the context for weighing the drug’s risks and benefits. For example, a drug intended to treat patients with a life-threatening disease for which no other therapy exists may be considered to have benefits that outweigh the risks even if those risks would be considered unacceptable for a condition that is not life threatening.
- *Assessment of benefits and risks from clinical data*—FDA reviewers evaluate clinical benefit and risk information submitted by the drug maker, taking into account any uncertainties that may result from imperfect or incomplete data. Generally, the agency expects that the drug maker will submit results from two well-designed clinical trials, to be sure that the findings from the first trial are not the result of chance or bias. In certain cases, especially if the disease is rare and multiple trials may not be feasible, convincing evidence from one clinical trial may be enough. Evidence that the drug will benefit the target population should outweigh any risks and uncertainties.

- *Strategies for managing risks*—All drugs have risks. Risk management strategies include an FDA-approved drug label, which clearly describes the drug's benefits and risks, and how the risks can be detected and managed. Sometimes, more effort is needed to manage risks. In these cases, a drug maker may need to implement a Risk Management and Mitigation Strategy (REMS).

Although many of the FDA's risk-benefit assessments and decisions are straightforward, sometimes the benefits and risks are uncertain and may be difficult to interpret or predict. The agency and the drug maker may reach different conclusions after analyzing the same data, or there may be differences of opinion among members of the FDA's review team. As a science-led organization, FDA uses the best scientific and technological information available to make decisions through a deliberative process.

Accelerated Approval

In some cases, the approval of a new drug is expedited. Accelerated Approval can be applied to promising therapies that treat a serious or life-threatening condition and provide therapeutic benefit over available therapies. This approach allows for the approval of a drug that demonstrates an effect on a "surrogate endpoint" that is reasonably likely to predict clinical benefit, or on a clinical endpoint that occurs earlier but may not be as robust as the standard endpoint used for approval. This approval pathway is especially useful when the drug is meant to treat a disease whose course is long, and an extended period of time is needed to measure its effect. After the drug enters the market, the drug maker is required to conduct post-marketing clinical trials to verify and describe the drug's benefit. If further trials fail to verify the predicted clinical benefit, FDA may withdraw approval.

Since the Accelerated Approval pathway was established in 1992, many drugs that treat life-threatening diseases have successfully been brought to market this way and have made a significant impact on disease course. For example, many antiretroviral drugs used to treat HIV/AIDS entered the market via accelerated approval, and subsequently altered the treatment paradigm. A number of targeted cancer-fighting drugs also have come onto the market through this pathway.

More information on Accelerated Approval is **here (/ForPatients/Approvals/Fast/ucm405447.htm)**.

Drug Development Designations

The agency also employs several approaches to encourage the development of certain drugs, especially drugs that may represent the first available treatment for an illness, or ones that have a significant benefit over existing drugs. These approaches, or designations, are meant to address specific needs, and a new drug application may receive more than one designation, if applicable. Each designation helps ensure that therapies for serious conditions are made available to patients as soon as reviewers can conclude that their benefits justify their risks.

- **Fast Track** is a process designed to facilitate the development and advance the review of drugs that treat serious conditions, and fill an unmet medical need, based on promising animal or human data. Fast tracking can get important new drugs to the patient earlier. The drug company must request the Fast Track process. More information about the Fast Track process is **here (/ForPatients/Approvals/Fast/ucm405399.htm)**.
- **Breakthrough Therapy** designation expedites the development and review of drugs that are intended to treat a serious condition, and preliminary clinical evidence indicates that the drug may demonstrate substantial improvement over available therapy. A drug with Breakthrough Therapy designation is also eligible for the Fast Track process. The drug company must request a Breakthrough Therapy designation. More information about Breakthrough Therapy designation is **here (/ForPatients/Approvals/Fast/ucm405397.htm)**.
- **Priority Review** means that FDA aims to take action on an application within six months, compared to 10 months under standard review. A Priority Review designation directs attention and resources to evaluate drugs that would significantly improve the treatment, diagnosis, or prevention of serious conditions. More information about Priority Review is **here (/ForPatients/Approvals/Fast/ucm405405.htm)**.

Spotlight

Step 1: Discovery and Development

Discovery

Typically, researchers discover new drugs through:

- New insights into a disease process that allow researchers to design a product to stop or reverse the effects of the disease.
- Many tests of molecular compounds to find possible beneficial effects against any of a large number of diseases.
- Existing treatments that have unanticipated effects.
- New technologies, such as those that provide new ways to target medical products to specific sites within the body or to manipulate genetic material.

At this stage in the process, thousands of compounds may be

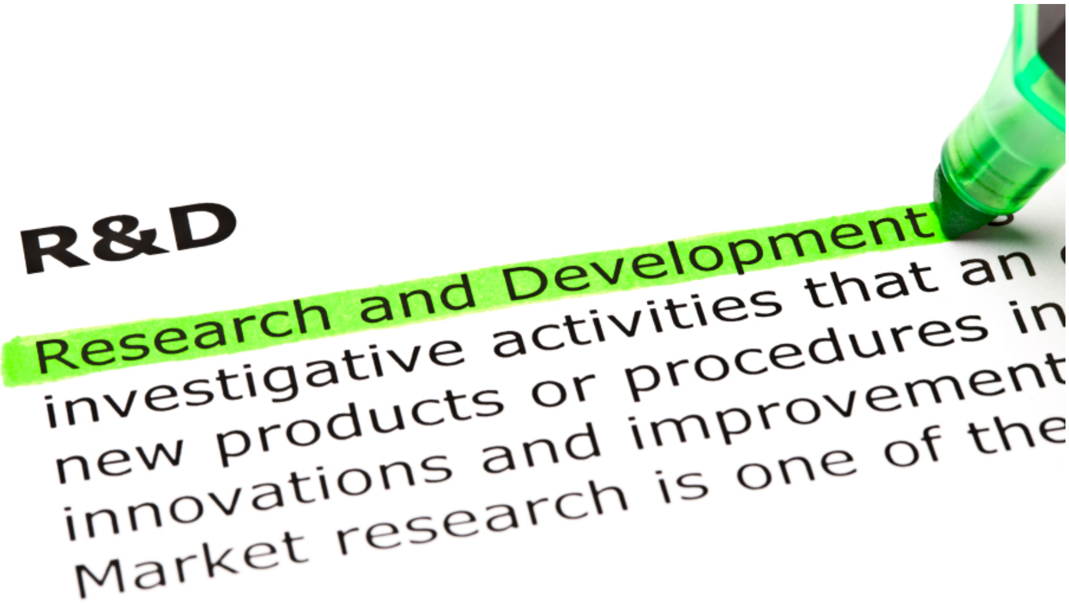

potential candidates for development as a medical treatment. After early testing, however, only a small number of compounds look promising and call for further study.

Development

Once researchers identify a promising compound for development, they conduct experiments to gather information on:

- How it is absorbed, distributed, metabolized, and excreted.
- Its potential benefits and mechanisms of action.
- The best dosage.
- The best way to give the drug (such as by mouth or injection).
- Side effects or adverse events that can often be referred to as toxicity.
- How it affects different groups of people (such as by gender, race, or ethnicity) differently.
- How it interacts with other drugs and treatments.
- Its effectiveness as compared with similar drugs.

"Image/Content by the United States Drug Administration, retrieved from: https://www.fda.gov/drugs/development-approval-process-drugs on August 30, 2017. Image/Content is in the public domain."
