# Supplementary material for: Pharmacology, Pharmacotherapy, and Pharmacopolicy Through an Evidence-Based Medicine: A Novel Approach for First-Year Medical Students
Source: MedEdPORTAL. 2020 Jul 20;16:10934. doi: 10.15766/mep_2374-8265.10934 (PMC7373350; doi:10.15766/mep_2374-8265.10934)
Supplement: Supplementary file 1 — Activity Information.docxUSDA QuickSheet.pdfFDA QuickSheet.pdfAdverse vs Side Effects.docxSeating Chart.pdfAcetaminophen Handout.pdfBeano Handout.docxMevacor Handout.pdfNaproxen Handout.pdfPraluent Handout.pdfXenical Handout.pdfFat-Soluble Vitamins Handout.pdfGroup Quiz.docxQuiz Answers.docx [file mep_2374-8265.10934-s001.zip › C. FDA QuickSheet.pdf]

U.S. Food and Drug Administration

Drug Approval Process

What is a drug as defined by the FDA?

A drug is any product that is intended for use in the diagnosis, cure mitigation, treatment , or prevention of disease; and that tis intended to affect the structure or any function of the body.

PRE-CLINICAL

Drug Sponsor's Discovery and Screening Phase

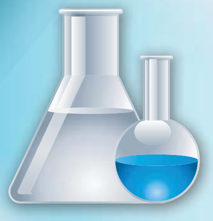

Drug Developed

Drug sponsor develops a new drug compound and seeks to have it approved by FDA for sale in the United States.

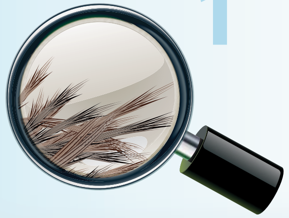

1

Animals Tested

Sponsor must test new drug on animals for toxicity. Multiple species are used to gather basic information on the safety and efficacy of the compound being investigated/researched.

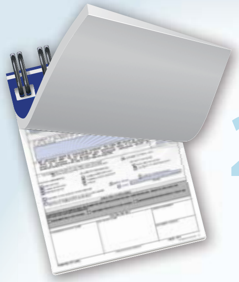

2

IND Application

The sponsor submits an Investigational New Drug (IND) application to FDA based on the results from intial testing that include, the drug's composition and manufacturing, and develops a plan for testing the drug on humans.

FDA's Center for Drug Evaluation and Research (CDER) evaluates new drugs before they can be sold.

The center's evaluation not only prevents quackery, but also provides doctors and patients the information they need to use medicines wisely. CDER ensures that drugs, both brand-name and generic, are effective and their health benefits outweigh their known risks.

IND REVIEW

FDA reviews the IND to assure that the proposed studies, generally referred to as clinical trials, do not place human subjects at unreasonable risk of harm. FDA also verifies that there are adequate informed consent and human subject protection.

CLINICAL

Drug Sponsor's Clinical Studies/Trials

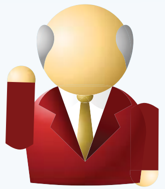

3

PHASE 1

20-80

The typical number of healthy volunteers used in Phase 1; this phase emphasizes safety. The goal here in this phase is to determine what the drug's most frequent side effects are and, often, how the drug is metabolized and excreted.

4

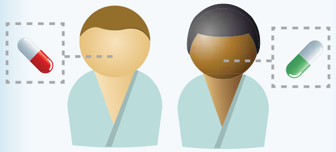

PHASE 2

100's

The typical number of patients used in Phase 2; this phase emphasizes effectiveness. This goal is to obtain preliminary data on whether the drug works in people who have a certain disease or condition. For controlled trials, patients receiving the drug are compared with similar patients receiving a different treatment--usually a placebo, or a different drug. Safety continues to be evaluated, and short-term side effects are studied.

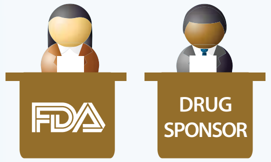

At the end of Phase 2, FDA and sponsors discuss how large-scale studies in Phase 3 will be done.

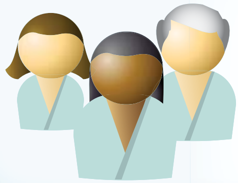

5

PHASE 3

1000's

The typical number of patients used in Phase 3. These studies gather more information about safety and effectiveness, study different populations and different dosages, and uses the drug in combination with other drugs.

Who reviews new drug submissions?

A team of CDER physicians, statisticians, chemists, pharmacologists, and other scientists review the drug sponsor's data and proposed labeling of drugs.

What other drug products are regulated by FDA?

Drugs include more than just medicines. For example, fluoride toothpastes, antiperspirants (not deodorant), dandruff shampoos, and sunscreens are all considered drugs.

NDA REVIEW

FDA's New Drug Application (NDA)Review

6

Review Meeting

FDA meets with a drug sponsor prior to submission of a New Drug Application.

7

NDA Application

The drug sponsor formally asks FDA to approve a drug for marketing in the United States by submitting an NDA. An NDA includes all animal and human data and analyses of the data, as well as information about how the drug behaves in the body and how it is manufactured.

8-9

Application Reviewed

After an NDA is received, FDA has 60 days to decide whether to file it so it can be reviewed. If FDA files the NDA, the FDA Review team is assigned to evaluate the sponsor's research on the drug's safety and effectiveness.

10

Drug Labeling

FDA reviews the drug's professional labeling and assures appropriate information is communicated to health care professionals and consumers.

11

Facility Inspection

FDA inspects the facilities where the drug will be manufactured.

12 FDA

Drug Approval

FDA reviewers will approve the application or issue a response letter.

FASTER APPROVALS

The **Accelerated Approval** program allows earlier approval of drugs that treat serious diseases and that fill an unmet medical need. The approval is faster because FDA can base the drug's effectiveness on a "surrogate endpoint," such as a blood test or X-ray result, rather than waiting for results from a clinical trial.

The **Fast Track** program helps reduce the time for FDA's review of products that treat serious or life-threatening diseases and those that have the potential to address an unmet medical need. Drug sponsors can submit portions of an application as the information becomes available ("rolling submission") instead of having to wait until all information is available.

POST-MARKETING

FDA's Post-Approval Risk Assessment Systems

PHASE 4

Because it's not possible to predict all of a drug's effects during clinical trials, monitoring safety issues after drugs get on the market is critical. The role of FDA's post-marketing safety system is to detect serious unexpected adverse events and take definitive action when needed.

Once FDA approves a drug, the post-marketing monitoring stage begins. The sponsor (typically the manufacturer) is required to submit periodic safety updates to FDA.

www.fda.gov/medwatch
(800) FDA-1088 (322-1088) phone
(800) FDA-0178 (322-0178) fax

MEDWATCH

FDA's MedWatch voluntary system makes it easier for physicians and consumers to report adverse events. Usually, when important new risks are uncovered, the risks are added to the drug's labeling and the public is informed of the new information through letters, public health advisories, and other education. In some cases, the use of the drug must be substantially limited. And in rare cases, the drug needs to be withdrawn from the market.

PDUFA

Prescription Drug User Fee Act

Since the PDUFA was passed in 1992, more than 1,000 drugs and biologics have come to the market, including new medicines to treat cancer, AIDS, cardiovascular disease, and life-threatening infections.

PDUFA has enabled the Food and Drug Administration to bring access to new drugs as fast or faster than anywhere in the world, all while maintaining the same thorough review process. Under PDUFA, drug companies agree to pay fees that boost FDA resources, and FDA agrees to time frames for its review of new drug applications.

Image/Content by the Federal Drug Administration, retrieved from: https://www.fda.gov/drugs/drug-information-consumers/fda-drug-approval-process-infographic-horizontal on August 30, 2017. Image/Content is in the public domain.
