# Supplementary material for: Pharmacology, Pharmacotherapy, and Pharmacopolicy Through an Evidence-Based Medicine: A Novel Approach for First-Year Medical Students
Source: MedEdPORTAL. 2020 Jul 20;16:10934. doi: 10.15766/mep_2374-8265.10934 (PMC7373350; doi:10.15766/mep_2374-8265.10934)
Supplement: Supplementary file 1 — Activity Information.docxUSDA QuickSheet.pdfFDA QuickSheet.pdfAdverse vs Side Effects.docxSeating Chart.pdfAcetaminophen Handout.pdfBeano Handout.docxMevacor Handout.pdfNaproxen Handout.pdfPraluent Handout.pdfXenical Handout.pdfFat-Soluble Vitamins Handout.pdfGroup Quiz.docxQuiz Answers.docx [file mep_2374-8265.10934-s001.zip › D. Adverse vs Side Effects.docx]

Adverse Effects v. Side Effects: Handout

**“Adverse Events**: Medical occurrence temporally associated with the use of a medicine or product, but not necessarily causally related.

**Adverse Reaction**: A response to a drug which is noxious and unintended, and which occurs at doses normally used in man for the prophylaxis, diagnosis, or therapy of disease, or for the modifications of physiological function.

**Side Effect**: Unintended, but rationalizable, effect occurring at normal dose related to the pharmacological properties of a medication.”

Reference:

Definitions. World Health Organization. <https://www.who.int/medicines/areas/quality_safety/safety_efficacy/trainingcourses/definitions.pdf>. Accessed 17 August 2019.
