# Supplementary material for: Pharmacology, Pharmacotherapy, and Pharmacopolicy Through an Evidence-Based Medicine: A Novel Approach for First-Year Medical Students
Source: MedEdPORTAL. 2020 Jul 20;16:10934. doi: 10.15766/mep_2374-8265.10934 (PMC7373350; doi:10.15766/mep_2374-8265.10934)
Supplement: Supplementary file 1 — Activity Information.docxUSDA QuickSheet.pdfFDA QuickSheet.pdfAdverse vs Side Effects.docxSeating Chart.pdfAcetaminophen Handout.pdfBeano Handout.docxMevacor Handout.pdfNaproxen Handout.pdfPraluent Handout.pdfXenical Handout.pdfFat-Soluble Vitamins Handout.pdfGroup Quiz.docxQuiz Answers.docx [file mep_2374-8265.10934-s001.zip › E. Seating Chart.pdf]

APPROXIMATE LOCATIONS OF COLOR-CODED GROUPS

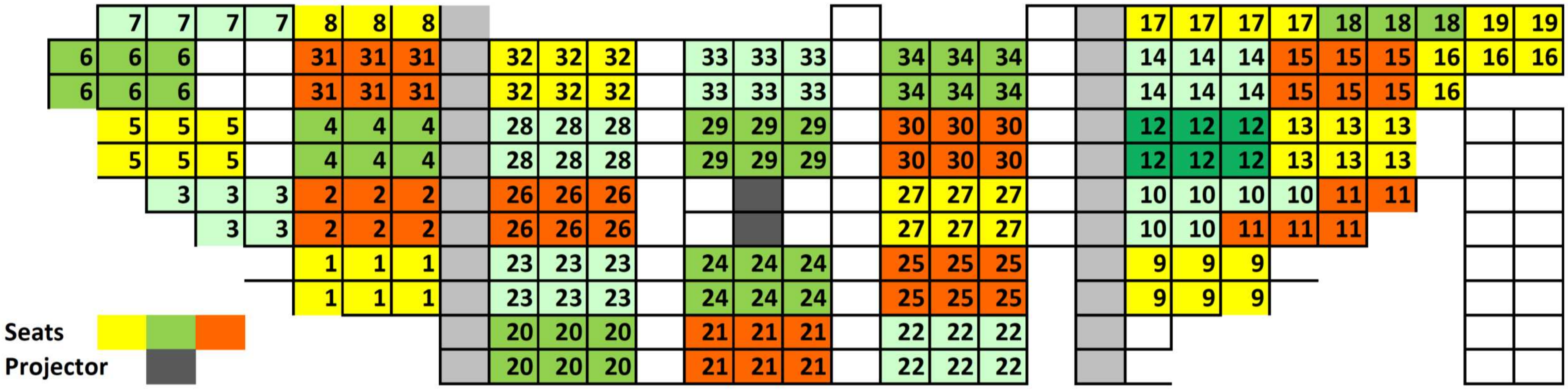

ORANGE

COLOR-CODE ORANGE	LOCATION
Groups 1-5	GROUP 1
Groups 6-10	GROUP 3
Groups 11-15	GROUP 4
Groups 16-20	GROUP 8
Groups 21-25	GROUP 8
Groups 26-30	GROUP 20
Groups 31-34	GROUP 26

GREEN

COLOR-CODE GREEN	LOCATION
Groups 1-5	GROUP 28
Groups 6-10	GROUP 32
Groups 11-15	GROUP 33
Groups 16-20	GROUP 29
Groups 21-25	GROUP 24
Groups 26-30	GROUP 22
Groups 31-34	GROUP 25

BLUE

COLOR_CODE BLUE	LOCATION
Groups 1-5	GROUP 30
Groups 6-10	GROUP 34
Groups 11-15	GROUP 9
Groups 16-20	GROUP 12
Groups 21-25	GROUP 17
Groups 26-30	GROUP 13
Groups 31-34	GROUP 14

Author owned.
