# Supplementary material for: Pharmacology, Pharmacotherapy, and Pharmacopolicy Through an Evidence-Based Medicine: A Novel Approach for First-Year Medical Students
Source: MedEdPORTAL. 2020 Jul 20;16:10934. doi: 10.15766/mep_2374-8265.10934 (PMC7373350; doi:10.15766/mep_2374-8265.10934)
Supplement: Supplementary file 1 — Activity Information.docxUSDA QuickSheet.pdfFDA QuickSheet.pdfAdverse vs Side Effects.docxSeating Chart.pdfAcetaminophen Handout.pdfBeano Handout.docxMevacor Handout.pdfNaproxen Handout.pdfPraluent Handout.pdfXenical Handout.pdfFat-Soluble Vitamins Handout.pdfGroup Quiz.docxQuiz Answers.docx [file mep_2374-8265.10934-s001.zip › F. Acetaminophen Handout.pdf]

HIGHLIGHTS OF PRESCRIBING INFORMATION
These highlights do not include all the information needed to use Acetaminophen Injection safely and effectively. See full prescribing information for Acetaminophen Injection.

Acetaminophen Injection, for intravenous use
Initial U.S. Approval: 1951

WARNING: RISK OF MEDICATION ERRORS AND HEPATOTOXICITY
See full prescribing information for complete boxed warning

Take care when prescribing, preparing, and administering Acetaminophen Injection to avoid dosing errors which could result in accidental overdose and death. (5.3)

Acetaminophen Injection contains acetaminophen. Acetaminophen has been associated with cases of acute liver failure, at times resulting in liver transplant and death. Most of the cases of liver injury are associated with the use of acetaminophen at doses that exceed the recommended maximum daily limits, and often involve more than one acetaminophen-containing product. *(5.1)*.

----------------------------INDICATIONS AND USAGE---------------------------
Acetaminophen injection is indicated for the:
- Management of mild to moderate pain. (1)
- Management of moderate to severe pain with adjunctive opioid analgesics. (1)
- Reduction of fever. (1)

----------------------DOSAGE AND ADMINISTRATION----------------------
- Acetaminophen injection may be given as a single or repeated dose. (2.1)
- Acetaminophen injection should be administered only as a 15 minute intravenous infusion. (2.4)

Adults and Adolescents Weighing 50 kg and Over:
- 1,000 mg every 6 hours or 650 mg every 4 hours to a maximum of 4,000 mg per day. Minimum dosing interval of 4 hours. (2.2)

Adults and Adolescents Weighing Under 50 kg:
- 15 mg/kg every 6 hours or 12.5 mg/kg every 4 hours to a maximum of 75 mg/kg per day. Minimum dosing interval of 4 hours. (2.2)

Children:
- Children 2 to 12 years of age: 15 mg/kg every 6 hours or 12.5 mg/kg every 4 hours to a maximum of 75 mg/kg per day. Minimum dosing interval of 4 hours. (2.3)

---------------------DOSAGE FORMS AND STRENGTHS---------------------
- Injection for intravenous infusion.
- Each 100 mL flexible plastic container has 1,000 mg acetaminophen (10 mg/mL). (3)

------------------------------CONTRAINDICATIONS-----------------------------
Acetaminophen is contraindicated:
- In patients with known hypersensitivity to acetaminophen or to any of the excipients in the IV formulation. (4)
- In patients with severe hepatic impairment or severe active liver disease. (4)

-----------------------WARNINGS AND PRECAUTIONS-----------------------
- Administration of acetaminophen in doses higher than recommended (by all routes of administration and from all acetaminophen-containing products including combination products) may result in hepatic injury, including the risk of liver failure and death. (5.1)
- Do not exceed the maximum recommended daily dose of acetaminophen (by all routes of administration and all acetaminophen-containing products including combination products). (5.1)
- Take care when prescribing, preparing, and administering acetaminophen injection to avoid dosing errors which could result in accidental overdose and death. (5.3)
- Use caution when administering acetaminophen in patients with the following conditions: hepatic impairment or active hepatic disease, in cases of alcoholism, chronic malnutrition, severe hypovolemia, or severe renal impairment (creatinine clearance ≤ 30 mL/min). (5.1)
- Discontinue acetaminophen immediately at the first appearance of skin rash and if symptoms associated with allergy or hypersensitivity occur. Do not use in patients with acetaminophen allergy. (5.2, 5.4)

------------------------------ADVERSE REACTIONS------------------------------
The most common adverse reactions in patients treated with acetaminophen were nausea, vomiting, headache, and insomnia in adult patients and nausea, vomiting, constipation, pruritus, agitation, and atelectasis in pediatric patients. (6.1)

To report SUSPECTED ADVERSE REACTIONS, contact Fresenius Kabi USA, LLC, Vigilance & Medical Affairs at 1-800-551-7176 or FDA at 1-800-FDA-1088 or www.fda.gov/medwatch.

------------------------------DRUG INTERACTIONS------------------------------
- Substances that induce or regulate hepatic cytochrome enzyme CYP2E1 may alter the metabolism of acetaminophen and increase its hepatotoxic potential. (7.1)
- Chronic oral acetaminophen use at a dose of 4,000 mg/day has been shown to cause an increase in international normalized ratio (INR) in some patients who have been stabilized on sodium warfarin as an anticoagulant. (7.2)

-----------------------USE IN SPECIFIC POPULATIONS-----------------------
- Pregnancy: Category C. There are no studies of intravenous acetaminophen in pregnant women. Use only if clearly needed. (8.1)
- Nursing Mothers: Caution should be exercised when administered to a nursing woman. (8.3)
- Pediatric Use: The effectiveness of acetaminophen for the treatment of acute pain and fever has not been studied in pediatric patients less than 2 years of age. The safety and effectiveness of acetaminophen in pediatric patients older than 2 years is supported by evidence from adequate and well-controlled studies in adults with additional safety and pharmacokinetic data for this age group. (8.4)
- Geriatric Use: No overall differences in safety or effectiveness were observed between geriatric and younger subjects. (8.5)
- Hepatic Impairment: Acetaminophen is contraindicated in patients with severe hepatic impairment or severe active liver disease and should be used with caution in patients with hepatic impairment or active liver disease. (4, 5.1, 8.6)
- Renal Impairment: In cases of severe renal impairment, longer dosing intervals and a reduced total daily dose of acetaminophen may be warranted. (5.1, 8.7)

Revised: 10/2015

Reference ID: 3839318

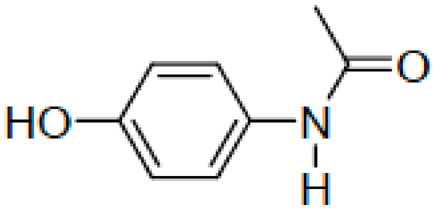

M.W. 151.16

Acetaminophen injection, for intravenous use, is a sterile, clear, colorless, non-pyrogenic, isotonic formulation of acetaminophen intended for intravenous infusion. It has a pH of approximately 5.5 and an osmolality of approximately 290 mOsm/kg. Each 100 mL contains acetaminophen, USP, 1,000 mg; mannitol, 3,670 mg and cysteine, 10 mg. pH is adjusted with hydrochloric acid and/or sodium hydroxide.

12 CLINICAL PHARMACOLOGY

12.1 Mechanism of Action

The precise mechanism of the analgesic and antipyretic properties of acetaminophen is not established but is thought to primarily involve central actions.

12.2 Pharmacodynamics

Acetaminophen has been shown to have analgesic and antipyretic activities in animal and human studies.

Single doses of acetaminophen up to 3,000 mg and repeated doses of 1,000 mg every 6 hours for 48 hours have not been shown to cause a significant effect on platelet aggregation. Acetaminophen does not have any immediate or delayed effects on small-vessel hemostasis. Clinical studies of both healthy subjects and patients with hemophilia showed no significant changes in bleeding time after receiving multiple doses of oral acetaminophen.

Image/Content by the Federal Drug Administration, retrieved from: https://www.accessdata.fda.gov/drugsatfda_docs/label/2015/204767s000lbl.pdf on August 30, 2017. Image/Content is in the public domain.
