# Supplementary material for: Pharmacology, Pharmacotherapy, and Pharmacopolicy Through an Evidence-Based Medicine: A Novel Approach for First-Year Medical Students
Source: MedEdPORTAL. 2020 Jul 20;16:10934. doi: 10.15766/mep_2374-8265.10934 (PMC7373350; doi:10.15766/mep_2374-8265.10934)
Supplement: Supplementary file 1 — Activity Information.docxUSDA QuickSheet.pdfFDA QuickSheet.pdfAdverse vs Side Effects.docxSeating Chart.pdfAcetaminophen Handout.pdfBeano Handout.docxMevacor Handout.pdfNaproxen Handout.pdfPraluent Handout.pdfXenical Handout.pdfFat-Soluble Vitamins Handout.pdfGroup Quiz.docxQuiz Answers.docx [file mep_2374-8265.10934-s001.zip › G. Beano Handout.docx]

Beano® Drug Information (Adapted for SIG3)

**“Product Facts**

Beano® contains a natural food enzyme that helps prevent gas before it starts. It works with your body’s digestion to break down the complex carbohydrates in gassy foods, like fresh vegetables, whole grain breads and beans, making them more digestible. Beano® enables you to enjoy your favorite healthy foods, whether at home, in a restaurant or at a friend’s house, without worrying about gas.

Beano® contains an enzyme from a natural source that works with your body’s digestion. It breaks down the complex carbohydrates found in gassy foods into simpler, easily digestible sugars before they reach the colon, preventing gas before it starts.

Beano® tablets contain less than five milligrams of sodium per tablet. It can be said that Beano® tablets are sodium-free.

Beano® tablets are available in the antacid section of supermarkets, pharmacies, mass merchandising outlets and health food stores across the country. Beano® is also available in outlets like Sam’s Club and Costco.

While there is no cure for lactose intolerance the natural enzyme in Beano® + Dairy Defense™ can help break down lactose preventing digestive disturbances.

Gluten is a protein found in wheat, rye, barley and oats. The product will not work on any protein, including gluten. This product is gluten free.

**Food & Gas**

Beano® is not just for beans. It works on a whole variety of gassy foods, including many vegetables like cabbage, broccoli and cauliflower, grains, cereals, nuts, seeds, whole-grain breads and many other foods.

The body does not digest and absorb some carbohydrates in the small intestine because of a shortage or absence of certain enzymes. This undigested food then passes from the small intestine to the large intestine where bacteria break down the food, producing gas. The most common symptoms of gas are flatulence, abdominal bloating and digestive discomfort. In essence, the body lacks the enzymes needed to break down the carbohydrates found in some gassy foods like vegetables, beans, grains, cereals, nuts, seeds and whole-grain breads. This is where Beano® steps in. Beano® contains a food enzyme from a natural source that works with your body’s digestion to break down the complex sugars in gassy foods making them more digestible, preventing gas before it even starts.

**Usage**

The correct timing of the dosage is also key to Beano®’s efficiency. To prevent gas before it starts, take Beano® with your gassy food. If you are using Beano® tablets, you should swallow, chew or crumble them onto your food right before your first bite or you will take with your first bite of food. If you are using Beano® Meltaways™, one Beano® Meltaway™ works the same as two Beano® tablets. Just place on your tongue before your first bite, and it will melt away in seconds with a smooth, light flavor to help prevent gas from happening.

The correct dosage amount is key to Beano®’s efficiency. Take 2 – 3 chewable tablets or 1 Meltaway™ at every typical meal. For best results, you may have to adjust the number of tablets or Meltaways™ according to the number of servings.

Beano® contains an enzyme from a natural source that works with your body’s digestion to break down the complex carbohydrates in healthy foods making them more digestible. Beano® is not a drug. It is a natural product that can help prevent gas before it starts. Beano® is recommended for adults and for children 12 years and older. Beano® is not recommended for use with infants or young children, except on the advice of a pediatrician. There is no information to indicate that Beano® is other than safe for use during pregnancy or during breast-feeding. However, Beano® has not been tested on pregnant or lactating women. Therefore, any pregnant or lactating woman should consult with her physician before using Beano®.

Beano® is recommended for children 12 years and older. Beano® is not recommended for use in children under 12, except on the advice of a pediatrician.

Beano® has only been tested for human consumption, so we have no data on using the product for pets.

**Medical Considerations**

There is no information to indicate that Beano® is unsafe for use during pregnancy or while breast-feeding. However, Beano® has not been tested on pregnant women. Therefore, we recommend that pregnant or breast-feeding women should speak to their physician before using Beano®.

Each serving of Beano® itself contributes an extremely small number of calories (less than five) to your diet and would be expected to have an insignificant effect on blood glucose. However, Beano® is a natural source food enzyme that works by breaking down indigestible sugars into simpler, more digestible sugars. As a result, it has been estimated that the use of Beano® will produce an additional two to six grams of carbohydrates for every 100 grams of food treated by Beano®. If you have concerns about the use of Beano®, we recommend that you speak with your physician.

Persons who are galactosemic should speak with their physician prior to using Beano®.

There is nothing in the scientific literature to suggest that individuals with an allergy to penicillin or molds would have an allergy to Beano®.

There are no known drug interactions. Since an enzyme will catalyze chemical reactions specific only to that enzyme, they are quite predictable. Alpha-galactosidase enzyme activity is specific to materials containing one or more alpha-1,6-galactopryanosyl groups in their structure.

**Expiration Dates**

Expiration dates are printed on the side of the bottles.

We can only assure the level of enzyme activity claimed through the expiration date printed on the package. Beyond the expiration date, enzyme activity may be less than the amount claimed, and the product may not be as effective as when its fresh. We recommend that the product be replaced when it is past the expiration date. We also recommend that Beano® be stored below 25 C OR 77 F. Avoid heat to protect product freshness.”

**Reference:**

FAQs. Beano. <https://www.beanogas.com/faqs/>. Updated 2019. Accessed 17 August 2019.
