# Supplementary material for: Pharmacology, Pharmacotherapy, and Pharmacopolicy Through an Evidence-Based Medicine: A Novel Approach for First-Year Medical Students
Source: MedEdPORTAL. 2020 Jul 20;16:10934. doi: 10.15766/mep_2374-8265.10934 (PMC7373350; doi:10.15766/mep_2374-8265.10934)
Supplement: Supplementary file 1 — Activity Information.docxUSDA QuickSheet.pdfFDA QuickSheet.pdfAdverse vs Side Effects.docxSeating Chart.pdfAcetaminophen Handout.pdfBeano Handout.docxMevacor Handout.pdfNaproxen Handout.pdfPraluent Handout.pdfXenical Handout.pdfFat-Soluble Vitamins Handout.pdfGroup Quiz.docxQuiz Answers.docx [file mep_2374-8265.10934-s001.zip › H. Mevacor Handout.pdf]

9844662

TABLETS

# MEVACOR®
## (LOVASTATIN)

### DESCRIPTION

MEVACOR* (Lovastatin) is a cholesterol lowering agent isolated from a strain of *Aspergillus terreus.* After oral ingestion, lovastatin, which is an inactive lactone, is hydrolyzed to the corresponding β-hydroxyacid form. This is a principal metabolite and an inhibitor of 3-hydroxy-3-methylglutaryl-coenzyme A (HMG-CoA) reductase. This enzyme catalyzes the conversion of HMG-CoA to mevalonate, which is an early and rate limiting step in the biosynthesis of cholesterol.

Lovastatin is [1*S*-[1α(*R**),3α,7β,8β(2*S**,4*S**), 8aβ]]-1,2,3,7, 8,8a-hexahydro-3,7-dimethyl-8-[2-(tetrahydro-4-hydroxy-6-oxo-2*H*-pyran-2-yl)ethyl]-1-naphthalenyl 2-methylbutanoate. The empirical formula of lovastatin is $C_{24}H_{36}O_5$ and its molecular weight is 404.55. Its structural formula is:

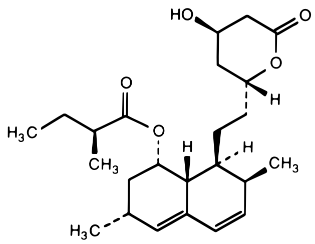


Lovastatin is a white, nonhygroscopic crystalline powder that is insoluble in water and sparingly soluble in ethanol, methanol, and acetonitrile.

Tablets MEVACOR are supplied as 20 mg and 40 mg tablets for oral administration. In addition to the active ingredient lovastatin, each tablet contains the following inactive ingredients: cellulose, lactose, magnesium stearate, and starch. Butylated hydroxyanisole (BHA) is added as a preservative. Tablets MEVACOR 20 mg also contain FD&C Blue 2 aluminum lake. Tablets MEVACOR 40 mg also contain D&C Yellow 10 aluminum lake and FD&C Blue 2 aluminum lake.

## CONTRAINDICATIONS

Hypersensitivity to any component of this medication.

Active liver disease or unexplained persistent elevations of serum transaminases (see WARNINGS).

Concomitant administration with strong CYP3A4 inhibitors (e.g., itraconazole, ketoconazole, posaconazole, HIV protease inhibitors, boceprevir, telaprevir, erythromycin, clarithromycin, telithromycin and nefazodone) (see WARNINGS, *Myopathy/Rhabdomyolysis)*.

*Pregnancy and lactation* (see PRECAUTIONS, *Pregnancy* and *Nursing Mothers*). Atherosclerosis is a chronic process and the discontinuation of lipid-lowering drugs during pregnancy should have little impact on the outcome of long-term therapy of primary hypercholesterolemia. Moreover, cholesterol and other products of the cholesterol biosynthesis pathway are essential components for fetal development, including synthesis of steroids and cell membranes. Because of the ability of inhibitors of HMG-CoA reductase such as MEVACOR to decrease the synthesis of cholesterol and possibly other products of the cholesterol biosynthesis pathway, MEVACOR is contraindicated during pregnancy and in nursing mothers. **MEVACOR should be administered to women of childbearing age only when such patients are highly unlikely to conceive.** If the patient becomes pregnant while taking this drug, MEVACOR should be discontinued immediately and the patient should be apprised of the potential hazard to the fetus (see PRECAUTIONS, *Pregnancy*).

## WARNINGS

### *Myopathy/Rhabdomyolysis*

Lovastatin, like other inhibitors of HMG-CoA reductase, occasionally causes myopathy manifested as muscle pain, tenderness or weakness with creatine kinase (CK) above ten times the upper limit of normal (ULN). Myopathy sometimes takes the form of rhabdomyolysis with or without acute renal failure secondary to myoglobinuria, and rare fatalities have occurred. The risk of myopathy is increased by high levels of HMG-CoA reductase inhibitory activity in plasma.

**As with other HMG-CoA reductase inhibitors, the risk of myopathy/rhabdomyolysis is dose related.** In a clinical study (EXCEL) in which patients were carefully monitored and some interacting drugs were excluded, there was one case of myopathy among 4933 patients randomized to lovastatin 20-40 mg daily for 48 weeks, and 4 among 1649 patients randomized to 80 mg daily.

**All patients starting therapy with lovastatin, or whose dose of lovastatin is being increased, should be advised of the risk of myopathy and told to report promptly any unexplained muscle pain, tenderness or weakness. Lovastatin therapy should be discontinued immediately if myopathy is diagnosed or suspected.** In most cases, muscle symptoms and CK increases resolved when treatment was promptly discontinued. Periodic CK determinations may be considered in patients starting therapy with lovastatin or whose dose is being increased, but there is no assurance that such monitoring will prevent myopathy.

Many of the patients who have developed rhabdomyolysis on therapy with lovastatin have had complicated medical histories, including renal insufficiency usually as a consequence of long-standing diabetes mellitus. Such patients merit closer monitoring. MEVACOR therapy should be discontinued if markedly elevated CPK levels occur or myopathy is diagnosed or suspected. MEVACOR therapy should also be temporarily withheld in any patient experiencing an acute or serious condition predisposing to the development of renal failure secondary to rhabdomyolysis, e.g., sepsis; hypotension; major surgery; trauma; severe metabolic, endocrine, or electrolyte disorders; or uncontrolled epilepsy.

**The risk of myopathy/rhabdomyolysis is increased by concomitant use of lovastatin with the following:**

**Strong inhibitors of CYP3A4:** Lovastatin, like several other inhibitors of HMG-CoA reductase, is a substrate of cytochrome P450 3A4 (CYP3A4). Certain drugs which inhibit this metabolic pathway can

raise the plasma levels of lovastatin and may increase the risk of myopathy. These include itraconazole, ketoconazole, and posaconazole, the macrolide antibiotics erythromycin and clarithromycin, and the ketolide antibiotic telithromycin, HIV protease inhibitors, boceprevir, telaprevir, or the antidepressant nefazodone. Combination of these drugs with lovastatin is contraindicated. If treatment with itraconazole, ketoconazole, posaconazole, erythromycin, clarithromycin or telithromycin is unavoidable, therapy with lovastatin should be suspended during the course of treatment (see CONTRAINDICATIONS; PRECAUTIONS, *Drug Interactions*).

Although not studied clinically, voriconazole has been shown to inhibit lovastatin metabolism *in vitro* (human liver microsomes). Therefore, voriconazole is likely to increase the plasma concentration of lovastatin. It is recommended that dose adjustment of lovastatin be considered during coadministration. Increased lovastatin concentration in plasma has been associated with an increased risk of myopathy/rhabdomyolysis.

**Gemfibrozil:** **The combined use of lovastatin with gemfibrozil should be avoided.**

**Other lipid-lowering drugs (other fibrates or ≥1 g/day of niacin):** Caution should be used when prescribing other fibrates or lipid-lowering doses (≥1 g/day) of niacin with lovastatin, as these agents can cause myopathy when given alone. **The benefit of further alterations in lipid levels by the combined use of lovastatin with other fibrates or niacin should be carefully weighed against the potential risks of these combinations.**

**Cyclosporine:** **The use of lovastatin with cyclosporine should be avoided.**

**Danazol, diltiazem or verapamil with higher doses of lovastatin:** **The dose of lovastatin should not exceed 20 mg daily in patients receiving concomitant medication with danazol, diltiazem, or verapamil.** The benefits of the use of lovastatin in patients receiving danazol, diltiazem, or verapamil should be carefully weighed against the risks of these combinations.

**Amiodarone:** **The dose of lovastatin should not exceed 40 mg daily in patients receiving concomitant medication with amiodarone.** The combined use of lovastatin at doses higher than 40 mg daily with amiodarone should be avoided unless the clinical benefit is likely to outweigh the increased risk of myopathy. The risk of myopathy/rhabdomyolysis is increased when amiodarone is used concomitantly with higher doses of a closely related member of the HMG-CoA reductase inhibitor class.

**Colchicine:** Cases of myopathy, including rhabdomyolysis, have been reported with lovastatin coadministered with colchicine, and caution should be exercised when prescribing lovastatin with colchicine (see PRECAUTIONS, *Drug Interactions*).

**Ranolazine:** The risk of myopathy, including rhabdomyolysis, may be increased by concomitant administration of ranolazine. Dose adjustment of lovastatin may be considered during co-administration with ranolazine.

Prescribing recommendations for interacting agents are summarized in Table VII (see also CLINICAL PHARMACOLOGY, *Pharmacokinetics*; PRECAUTIONS, *Drug Interactions*; DOSAGE AND ADMINISTRATION).



Reference ID: 3093010

Table VII
Drug Interactions Associated with Increased
Risk of Myopathy/Rhabdomyolysis

| Interacting Agents | Prescribing Recommendations |
|---|---|
| Strong CYP3A4 inhibitors, e.g.:<br>Ketoconazole<br>Itraconazole<br>Posaconazole<br>Erythromycin<br>Clarithromycin<br>Telithromycin<br>HIV protease inhibitors<br>Boceprevir<br>Telaprevir<br>Nefazodone | Contraindicated with lovastatin |
| Gemfibrozil<br>Cyclosporine | Avoid with lovastatin |
| Danazol<br>Diltiazem<br>Verapamil | Do not exceed 20 mg lovastatin daily |
| Amiodarone | Do not exceed 40 mg lovastatin daily |
| Grapefruit juice | Avoid large quantities of grapefruit juice (>1 quart daily) |

*Liver Dysfunction*

**Persistent increases (to more than 3 times the upper limit of normal) in serum transaminases occurred in 1.9% of adult patients who received lovastatin for at least one year in early clinical trials (see ADVERSE REACTIONS).** When the drug was interrupted or discontinued in these patients, the transaminase levels usually fell slowly to pretreatment levels. The increases usually appeared 3 to 12 months after the start of therapy with lovastatin, and were not associated with jaundice or other clinical signs or symptoms. There was no evidence of hypersensitivity. In the EXCEL study (see CLINICAL PHARMACOLOGY, *Clinical Studies*), the incidence of persistent increases in serum transaminases over 48 weeks was 0.1% for placebo, 0.1% at 20 mg/day, 0.9% at 40 mg/day, and 1.5% at 80 mg/day in patients on lovastatin. However, in post-marketing experience with MEVACOR, symptomatic liver disease has been reported rarely at all dosages (see ADVERSE REACTIONS).

In AFCAPS/TexCAPS, the number of participants with consecutive elevations of either alanine aminotransferase (ALT) or aspartate aminotransferase (AST) (> 3 times the upper limit of normal), over a median of 5.1 years of follow-up, was not significantly different between the MEVACOR and placebo groups (18 [0.6%] vs. 11 [0.3%]). The starting dose of MEVACOR was 20 mg/day; 50% of the MEVACOR treated participants were titrated to 40 mg/day at Week 18. Of the 18 participants on MEVACOR with consecutive elevations of either ALT or AST, 11 (0.7%) elevations occurred in participants taking 20 mg/day, while 7 (0.4%) elevations occurred in participants titrated to 40 mg/day. Elevated transaminases resulted in discontinuation of 6 (0.2%) participants from therapy in the MEVACOR group (n=3,304) and 4 (0.1%) in the placebo group (n=3,301).

It is recommended that liver enzyme tests be obtained prior to initiating therapy with MEVACOR and repeated as clinically indicated.

There have been rare postmarketing reports of fatal and non-fatal hepatic failure in patients taking statins, including lovastatin. If serious liver injury with clinical symptoms and/or hyperbilirubinemia or jaundice occurs during treatment with MEVACOR, promptly interrupt therapy. If an alternate etiology is not found do not restart MEVACOR.

The drug should be used with caution in patients who consume substantial quantities of alcohol and/or have a past history of liver disease. Active liver disease or unexplained transaminase elevations are contraindications to the use of lovastatin.

As with other lipid-lowering agents, moderate (less than three times the upper limit of normal) elevations of serum transaminases have been reported following therapy with MEVACOR (see ADVERSE REACTIONS). These changes appeared soon after initiation of therapy with MEVACOR, were often transient, were not accompanied by any symptoms and interruption of treatment was not required.



Reference ID: 3093010

**PRECAUTIONS**

*General*

Lovastatin may elevate creatine phosphokinase and transaminase levels (see WARNINGS and ADVERSE REACTIONS). This should be considered in the differential diagnosis of chest pain in a patient on therapy with lovastatin.

*Homozygous Familial Hypercholesterolemia*

MEVACOR is less effective in patients with the rare homozygous familial hypercholesterolemia, possibly because these patients have no functional LDL receptors. MEVACOR appears to be more likely to raise serum transaminases (see ADVERSE REACTIONS) in these homozygous patients.

***Information for Patients***

**Patients should be advised about substances they should not take concomitantly with lovastatin and be advised to report promptly unexplained muscle pain, tenderness, or weakness (see list below and WARNINGS, *Myopathy/Rhabdomyolysis*). Patients should also be advised to inform other physicians prescribing a new medication that they are taking MEVACOR.**

It is recommended that liver enzymes be checked before starting therapy, and if signs or symptoms of liver injury occur. All patients treated with MEVACOR should be advised to report promptly any symptoms that may indicate liver injury, including fatigue, anorexia, right upper abdominal discomfort, dark urine or jaundice.

*Drug Interactions*

*CYP3A4 Interactions*

Lovastatin is metabolized by CYP3A4 but has no CYP3A4 inhibitory activity; therefore it is not expected to affect the plasma concentrations of other drugs metabolized by CYP3A4. Strong inhibitors of CYP3A4 (e.g., itraconazole, ketoconazole, posaconazole, clarithromycin, telithromycin, HIV protease inhibitors, boceprevir, telaprevir, nefazodone, and erythromycin), and large quantities of grapefruit juice (>1 quart daily) increase the risk of myopathy by reducing the elimination of lovastatin. **(See CONTRAINDICATIONS, WARNINGS, *Myopathy/Rhabdomyolysis*, and CLINICAL PHARMACOLOGY, *Pharmacokinetics.*)**

*In vitro* studies have demonstrated that voriconazole inhibits the metabolism of lovastatin. Adjustment of the lovastatin dose may be needed to reduce the risk of myopathy, including rhabdomyolysis, if voriconazole must be used concomitantly with lovastatin.

*Interactions With Lipid-Lowering Drugs That Can Cause Myopathy When Given Alone*

The risk of myopathy is also increased by the following lipid-lowering drugs that are not strong CYP3A4 inhibitors, but which can cause myopathy when given alone.

**See WARNINGS, *Myopathy/Rhabdomyolysis.***
**Gemfibrozil**
**Other fibrates**
**Niacin (nicotinic acid) (≥1 g/day)**

*Other Drug Interactions*

*Cyclosporine:* The risk of myopathy/rhabdomyolysis is increased by concomitant administration of cyclosporine (see WARNINGS, *Myopathy/Rhabdomyolysis*).

*Danazol, Diltiazem, or Verapamil:* The risk of myopathy/rhabdomyolysis is increased by concomitant administration of danazol, diltiazem, or verapamil particularly with higher doses of lovastatin (see WARNINGS, *Myopathy/Rhabdomyolysis*; CLINICAL PHARMACOLOGY, *Pharmacokinetics*).

*Amiodarone*: The risk of myopathy/rhabdomyolysis is increased when amiodarone is used concomitantly with a closely related member of the HMG-CoA reductase inhibitor class (see WARNINGS, *Myopathy/Rhabdomyolysis*).

*Coumarin Anticoagulants:* In a small clinical trial in which lovastatin was administered to warfarin treated patients, no effect on prothrombin time was detected. However, another HMG-CoA reductase inhibitor has been found to produce a less than two-second increase in prothrombin time in healthy

volunteers receiving low doses of warfarin. Also, bleeding and/or increased prothrombin time have been reported in a few patients taking coumarin anticoagulants concomitantly with lovastatin. It is recommended that in patients taking anticoagulants, prothrombin time be determined before starting lovastatin and frequently enough during early therapy to insure that no significant alteration of prothrombin time occurs. Once a stable prothrombin time has been documented, prothrombin times can be monitored at the intervals usually recommended for patients on coumarin anticoagulants. If the dose of lovastatin is changed, the same procedure should be repeated. Lovastatin therapy has not been associated with bleeding or with changes in prothrombin time in patients not taking anticoagulants.

*Colchicine:* Cases of myopathy, including rhabdomyolysis, have been reported with lovastatin coadministered with colchicine. See WARNINGS, *Myopathy/Rhabdomyolysis.*

*Ranolazine:* The risk of myopathy, including rhabdomyolysis, may be increased by concomitant administration of ranolazine. See WARNINGS, *Myopathy/Rhabdomyolysis*.

*Propranolol:* In normal volunteers, there was no clinically significant pharmacokinetic or pharmacodynamic interaction with concomitant administration of single doses of lovastatin and propranolol.

*Digoxin:* In patients with hypercholesterolemia, concomitant administration of lovastatin and digoxin resulted in no effect on digoxin plasma concentrations.

*Oral Hypoglycemic Agents:* In pharmacokinetic studies of MEVACOR in hypercholesterolemic non-insulin dependent diabetic patients, there was no drug interaction with glipizide or with chlorpropamide (see CLINICAL PHARMACOLOGY, *Clinical Studies*).

*Endocrine Function*

Increases in HbA1c and fasting serum glucose levels have been reported with HMG-CoA reductase inhibitors, including MEVACOR.

HMG-CoA reductase inhibitors interfere with cholesterol synthesis and as such might theoretically blunt adrenal and/or gonadal steroid production. Results of clinical trials with drugs in this class have been inconsistent with regard to drug effects on basal and reserve steroid levels. However, clinical studies have shown that lovastatin does not reduce basal plasma cortisol concentration or impair adrenal reserve, and does not reduce basal plasma testosterone concentration. Another HMG-CoA reductase inhibitor has been shown to reduce the plasma testosterone response to HCG. In the same study, the mean testosterone response to HCG was slightly but not significantly reduced after treatment with lovastatin 40 mg daily for 16 weeks in 21 men. The effects of HMG-CoA reductase inhibitors on male fertility have not been studied in adequate numbers of male patients. The effects, if any, on the pituitary-gonadal axis in pre-menopausal women are unknown. Patients treated with lovastatin who develop clinical evidence of endocrine dysfunction should be evaluated appropriately. Caution should also be exercised if an HMG-CoA reductase inhibitor or other agent used to lower cholesterol levels is administered to patients also receiving other drugs (e.g., spironolactone, cimetidine) that may decrease the levels or activity of endogenous steroid hormones.

*CNS Toxicity*

Lovastatin produced optic nerve degeneration (Wallerian degeneration of retinogeniculate fibers) in clinically normal dogs in a dose-dependent fashion starting at 60 mg/kg/day, a dose that produced mean plasma drug levels about 30 times higher than the mean drug level in humans taking the highest recommended dose (as measured by total enzyme inhibitory activity). Vestibulocochlear Wallerian-like degeneration and retinal ganglion cell chromatolysis were also seen in dogs treated for 14 weeks at 180 mg/kg/day, a dose which resulted in a mean plasma drug level ($C_{max}$) similar to that seen with the 60 mg/kg/day dose.

CNS vascular lesions, characterized by perivascular hemorrhage and edema, mononuclear cell infiltration of perivascular spaces, perivascular fibrin deposits and necrosis of small vessels, were seen in dogs treated with lovastatin at a dose of 180 mg/kg/day, a dose which produced plasma drug levels ($C_{max}$) which were about 30 times higher than the mean values in humans taking 80 mg/day.

Similar optic nerve and CNS vascular lesions have been observed with other drugs of this class.

Cataracts were seen in dogs treated for 11 and 28 weeks at 180 mg/kg/day and 1 year at 60 mg/kg/day.

Image/Content by the Federal Drug Administration, retrieved from: https://www.accessdata.fda.gov/drugsatfda_docs/label/2012/019643s085lbl.pdf on August 30, 2017. Image/Content is in the public domain.
