# Supplementary material for: Pharmacology, Pharmacotherapy, and Pharmacopolicy Through an Evidence-Based Medicine: A Novel Approach for First-Year Medical Students
Source: MedEdPORTAL. 2020 Jul 20;16:10934. doi: 10.15766/mep_2374-8265.10934 (PMC7373350; doi:10.15766/mep_2374-8265.10934)
Supplement: Supplementary file 1 — Activity Information.docxUSDA QuickSheet.pdfFDA QuickSheet.pdfAdverse vs Side Effects.docxSeating Chart.pdfAcetaminophen Handout.pdfBeano Handout.docxMevacor Handout.pdfNaproxen Handout.pdfPraluent Handout.pdfXenical Handout.pdfFat-Soluble Vitamins Handout.pdfGroup Quiz.docxQuiz Answers.docx [file mep_2374-8265.10934-s001.zip › I. Naproxen Handout.pdf]

Abridged for SIG 3

Naproxen

Dosage Form: oral suspension

Rx only

WARNING: RISK OF SERIOUS CARDIOVASCULAR AND GASTROINTESTINAL EVENTS

Cardiovascular Thrombotic Events

- Nonsteroidal anti-inflammatory drugs (NSAIDs) cause an increased risk of serious cardiovascular thrombotic events, including myocardial infarction and stroke, which can be fatal. This risk may occur early in treatment and may increase with duration of use *(see WARNINGS)*.
- Naproxen is contraindicated in the setting of coronary artery bypass graft (CABG) surgery *(see CONTRAINDICATIONS, WARNINGS).*

Gastrointestinal Bleeding, Ulceration, and Perforation

- NSAIDs cause an increased risk of serious gastrointestinal (GI) adverse events including bleeding, ulceration, and perforation of the stomach or intestines, which can be fatal. These events can occur at any time during use and without warning symptoms. Elderly patients and patients with a prior history of peptic ulcer disease and/or GI bleeding are at greater risk for serious GI events *(see WARNINGS).*

Naproxen Description

Naproxen is a proprionic acid derivative related to the arylacetic acid group of nonsteroidal anti-inflammatory drugs.

The chemical name for Naproxen is 2-naphthaleneacetic acid (s) 6-methoxy-a-methyl. It has the following structure:

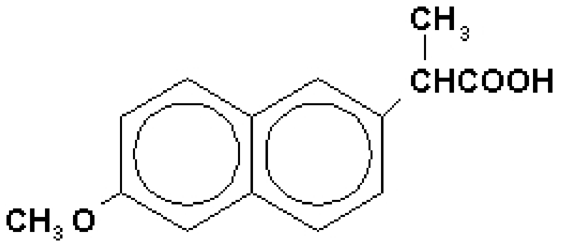

$C_{14}H_{14}O_3$ M.W. 230.26

Naproxen - Clinical Pharmacology

Mechanism of Action

Naproxen has analgesic, anti-inflammatory and antipyretic properties.

The mechanism of action of Naproxen, like that of other NSAIDs, is not completely understood but involves inhibition of cyclooxygenase (COX-1 and COX-2).

Naproxen is a potent inhibitor of prostaglandin synthesis in vitro. Naproxen concentrations reached during therapy have produced in vivo effects. Prostaglandins sensitize afferent nerves and potentiate the action of bradykinin in inducing pain in animal models. Prostaglandins are mediators of inflammation. Because Naproxen is an inhibitor of prostaglandin synthesis, its mode of action may be due to a decrease of prostaglandins in peripheral tissues.

Contraindications

Naproxen is contraindicated in the following patients:

- Known hypersensitivity (e.g., anaphylactic reactions and serious skin reactions) to Naproxen or any components of the drug product *(see WARNINGS: Anaphylactic Reactions, Serious Skin Reactions)*.

- History of asthma, urticaria, or other allergic-type reactions after taking aspirin or other NSAIDs. Severe, sometimes fatal, anaphylactic-reactions to NSAIDs have been reported in such patients *(see WARNINGS: Anaphylactic Reactions, Exacerbation of Asthma Related to Aspirin Sensitivity).*

- In the setting of coronary artery bypass graft (CABG) surgery (*see WARNINGS: Cardiovascular Thrombotic Events*).

Warnings

Cardiovascular Thrombotic Events

Clinical trials of several COX-2 selective and nonselective NSAIDs of up to three years duration have shown an increased risk of serious cardiovascular (CV) thrombotic events, including myocardial infarction (MI), and stroke, which can be fatal. Based on available data, it is unclear that the risk for CV thrombotic events is similar for all NSAIDs. The relative increase in serious CV thrombotic events over baseline conferred by NSAID use appears to be similar in those with and without known CV disease or risk factors for CV disease. However, patients with known CV disease or risk factors had a higher absolute incidence of excess serious CV thrombotic events, due to their increased baseline rate. Some observational studies found that this increased risk of serious CV thrombotic events began as early as the first weeks of treatment. The increase in CV thrombotic risk has been observed most consistently at higher doses.

To minimize the potential risk for an adverse CV event in NSAID-treated patients, use the lowest effective dose for the shortest duration possible. Physicians and patients should remain alert for the development of such events, throughout the entire treatment course, even in the absence of previous CV symptoms. Patients should be informed about the symptoms of serious CV events and the steps to take if they occur.

There is no consistent evidence that concurrent use of aspirin mitigates the increased risk of serious CV thrombotic events associated with NSAID use. The concurrent use of aspirin and an NSAID, such as Naproxen, increases the risk of serious gastrointestinal (GI) events *(see WARNINGS: Gastrointestinal, Bleeding, Ulceration and Perforation).*

Status Post Coronary Artery Bypass Graft (CABG) Surgery
Two large, controlled, clinical trials of a COX-2 selective NSAID for the treatment of pain in the first 10 to 14 days following CABG surgery found an increased incidence of myocardial infarction and stroke. NSAIDs are contraindicated in the setting of CABG *(see CONTRAINDICATIONS).*

Post-MI Patients
Observational studies conducted in the Danish National Registry have demonstrated that patients treated with NSAIDs in the post-MI period were at increased risk of reinfarction, CV-related death, and all-cause mortality beginning in the first week of treatment. In this same cohort, the incidence of death in the first year post MI was 20 per 100 person years in NSAID-treated patients compared to 12 per 100 person years in non-NSAID exposed patients. Although the absolute rate of death declined somewhat after the first year post-MI, the increased relative risk of death in NSAID users persisted over at least the next 4 years of follow-up.

Avoid the use of Naproxen in patients with a recent MI unless the benefits are expected to outweigh the risk of recurrent CV thrombotic events. If Naproxen is used in patients with a recent MI, monitor patients for signs of cardiac ischemia.

Gastrointestinal, Bleeding, Ulceration and Perforation

NSAIDs, including Naproxen, can cause serious gastrointestinal (GI) adverse events including inflammation, bleeding, ulceration, and perforation of the esophagus, stomach, small intestine, or large intestine, which can be fatal.

These serious adverse events can occur at any time, with or without warning symptoms, in patients treated with NSAIDs. Only one in five patients, who develop a serious upper GI adverse event on NSAID therapy, is symptomatic. Upper GI ulcers, gross bleeding, or perforation caused by NSAIDs occur in approximately 1% of patients treated for 3 to 6 months, and in about 2% to 4% of patients treated for one year. However, even short-term NSAID therapy is not without risk.

Risk Factors for GI Bleeding, Ulceration, and Perforation
Patients with a prior history of peptic ulcer disease and/or GI bleeding who used NSAIDs had a greater than 10-fold increased risk of developing a GI bleed compared to patients without these risk factors. Other factors that increase the risk of GI bleeding in patients treated with NSAIDs include longer duration of NSAID therapy; concomitant use of oral corticosteroids, aspirin, or anticoagulants, or selective serotonin reuptake inhibitors (SSRIs), smoking, use of alcohol, older age, and poor general health status. Most postmarketing reports of fatal GI events occurred in elderly or debilitated patients. Additionally, patients with advanced liver disease and/or coagulopathy are at increased risk for GI bleeding.

Strategies to Minimize the GI Risks in NSAID-treated patients:

- Use the lowest effective dosage for the shortest possible duration.
- Avoid administration of more than one NSAID at a time.
- Avoid use in patients at higher risk unless benefits are expected to outweigh the increased risk of bleeding. For such patients, as well as those with active GI bleeding, consider alternate therapies other than NSAIDs.
- Remain alert for signs and symptoms of GI ulceration and bleeding during NSAID therapy.
- If a serious GI adverse event is suspected, promptly initiate evaluation and treatment, and discontinue Naproxen until a serious GI adverse event is ruled out.
- In the setting of concomitant use of low-dose aspirin for cardiac prophylaxis, monitor patients more closely for evidence of GI bleeding *(see PRECAUTIONS: Drug Interactions).*

Hepatotoxicity

Elevations of ALT or AST (three or more times the upper limit of normal [ULN]) have been reported in approximately 1% of patients in clinical trials. In addition, rare, sometimes fatal, cases of severe hepatic injury, including fulminant hepatitis, liver necrosis and hepatic failure have been reported.

Elevations of ALT or AST (less than three times ULN) may occur in up to 15% of patients taking NSAIDs including Naproxen.

Inform patients of the warning signs and symptoms of hepatotoxicity (e.g., nausea, fatigue, lethargy, diarrhea, pruritus, jaundice, right upper quadrant tenderness, and "flu-like" symptoms). If clinical signs and symptoms consistent with liver disease develop, or if systemic manifestations occur (e.g., eosinophilia, rash, etc.), discontinue Naproxen immediately, and perform a clinical evaluation of the patient.

Hypertension

NSAIDs, including Naproxen, can lead to new onset of hypertension or worsening of pre-existing hypertension, either of which may contribute to the increased incidence of CV events. Patients taking angiotensin converting enzyme (ACE) inhibitors, thiazide diuretics, or loop diuretics may have impaired response to these therapies when taking NSAIDs (see PRECAUTIONS: Drug Interactions).

Monitor blood pressure (BP) during the initiation of NSAID treatment and throughout the course of therapy.

Heart Failure and Edema

The Coxib and traditional NSAID Trialists' Collaboration meta-analysis of randomized controlled trials demonstrated an approximately two-fold increase in hospitalizations for heart failure in COX-2 selective-treated patients and nonselective NSAID-treated patients compared to placebo-treated patients. In a Danish National Registry study of patients with heart failure, NSAID use increased the risk of MI, hospitalization for heart failure, and death.

Additionally, fluid retention, and edema have been observed in some patients treated with NSAIDs. Use of Naproxen may blunt the CV effects of several therapeutic agents used to treat these medical conditions [e.g., diuretics, ACE inhibitors, or angiotensin receptor blockers (ARBs)] *(see PRECAUTION: Drug Interactions)*.

Avoid the use of Naproxen in patients with severe heart failure unless the benefits are expected to outweigh the risk ofworsening heart failure. If Naproxen is used in patients with severe heart failure, monitor patients for signs of worsening heart failure.

Each 5 mL of Naproxen suspension contains 39 mg of sodium. This should be considered in patients whose overall intake of sodium must be severely restricted.

Renal Toxicity and Hyperkalemia

Long-term administration of NSAIDs has resulted in renal papillary necrosis and other renal injury. Renal toxicity has also been seen in patients in whom renal prostaglandins have a compensatory role in the maintenance of renal perfusion. In these patients, administration of an NSAID may cause a dose-dependent reduction in prostaglandin formation and, secondarily, in renal blood flow, which may precipitate overt renal decompensation. Patients at greatest risk of this reaction are those with impaired renal function, dehydration, hypovolemia, heart failure, liver dysfunction, those taking diuretics and ACE inhibitors or ARBs, and the elderly. Discontinuation of NSAID therapy is usually followed by recovery to the pretreatment state.

No information is available from controlled clinical studies regarding the use of Naproxen in patients with advanced renal disease. The renal effects of Naproxen may hasten the progression of renal dysfunction in patients with pre-existing renal disease.

Correct volume status in dehydrated or hypovolemic patients prior to initiating Naproxen. Monitor renal function in patients with renal or hepatic impairment, heart failure, dehydration, or hypovolemia during use of Naproxen *(see PRECAUTIONS: Drug Interactions)*. Avoid the use of Naproxen in patients with advanced renal disease unless the benefits are expected to outweigh the risk of worsening renal function. If Naproxen is used in patients with advanced renal disease, monitor patients for signs of worsening renal function.

Hyperkalemia

Increases in serum potassium concentration, including hyperkalemia, have been reported with use of NSAIDs, even in some patients without renal impairment. In patients with normal renal function, these effects have been attributed to a hyporeninemic-hypoaldosteronism state.

Anaphylactic Reactions

Naproxen has been associated with anaphylactic reactions in patients with and without known hypersensitivity to Naproxen and in patients with aspirin-sensitive asthma (see CONTRAINDICATIONS, WARNINGS: Exacerbation of Asthma Related to Aspirin Sensitivity).

Exacerbation of Asthma Related to Aspirin Sensitivity

A subpopulation of patients with asthma may have aspirin-sensitive asthma which may include chronic rhinosinusitis complicated by nasal polyps; severe, potentially fatal bronchospasm; and/or intolerance to aspirin and other NSAIDs. Because cross-reactivity between aspirin and other NSAIDs has been reported in such aspirin-sensitive patients, Naproxen is contraindicated in patients with this form of aspirin sensitivity (see CONTRAINDICATIONS). When Naproxen is used in patients with preexisting asthma (without known aspirin sensitivity), monitor patients for changes in the signs and symptoms of asthma.

Serious Skin Reactions

NSAIDs, including Naproxen, can cause serious skin adverse reactions such as exfoliative dermatitis, Stevens-Johnson Syndrome (SJS), and toxic epidermal necrolysis (TEN), which can be fatal. These serious events may occur without warning. Inform patients about the signs and symptoms of serious skin reactions and to discontinue the use of Naproxen at the first appearance of skin rash or any other sign of hypersensitivity. Naproxen is contraindicated in patients with previous serious skin reactions to NSAIDs *(see CONTRAINDICATIONS)*.

Premature Closure of Fetal Ductus Arteriosus

Naproxen may cause premature closure of the fetal ductus arteriosus. Avoid use of NSAIDs, including Naproxen, in pregnant women starting at 30 weeks of gestation (third trimester) *(seePRECAUTIONS: Pregnancy)*.

Hematologic Toxicity

Anemia has occurred in NSAID-treated patients. This may be due to occult or gross blood loss, fluid retention, or an incompletely described effect on erythropoiesis. If a patient treated with Naproxen has any signs or symptoms of anemia, monitor hemoglobin or hematocrit.

NSAIDs, including Naproxen, may increase the risk of bleeding events. Co-morbid conditions such as coagulation disorders, or concomitant use of warfarin, other anticoagulants, antiplatelet agents (e.g., aspirin), serotonin reuptake inhibitors (SSRIs) and serotonin norepinephrine reuptake inhibitors (SNRIs) may increase this risk. Monitor these patients for signs of bleeding *(see PRECAUTIONS: Drug Interactions)*.

Drug Interactions

See Table 1 for clinically significant drug interactions with Naproxen.

Table 1: Clinically Significant Drug Interactions with Naproxen

**Drugs That Interfere with Hemostasis**	
Clinical Impact:	•Naproxen and anticoagulants such as warfarin have a synergistic effect on bleeding. The concomitant use of Naproxen and anticoagulants have an increased risk of serious bleeding compared to the use of either drug alone.<br>•Serotonin release by platelets plays an important role in hemostasis. Case-control and cohort epidemiological studies showed that concomitant use of drugs that interfere with serotonin reuptake and an NSAID may potentiate the risk of bleeding more than an NSAID alone.
Intervention:	Monitor patients with concomitant use of Naproxen with anticoagulants (e.g., warfarin-ephrine reuptake inhibitors (SNRIs)) for signs of bleeding ***(see** WARNINGS: Hematologic Toxicity**)***.
**Aspirin**	
Clinical Impact:	Controlled clinical studies showed that the concomitant use of NSAIDs and analgesic doses of aspirin does not produce any greater therapeutic effect than the use of NSAIDs alone. In a clinical study, the concomitant use of an NSAID and aspirin was associated with a significantly increased incidence of GI adverse reactions as compared to use of the NSAID alone ***(see** WARNINGS: Gastrointestinal Bleeding, Ulceration and Perforation**)***.
Intervention:	Concomitant use of Naproxen and analgesic doses of aspirin is not generally recommended because of the increased risk of bleeding ***(see** WARNINGS: Hematologic Toxicity**).***<br>Naproxen is not a substitute for low dose aspirin for cardiovascular protection.
**ACE Inhibitors, Angiotensin Receptor Blockers, and Beta-Blockers**	

Clinical Impact:	• NSAIDs may diminish the antihypertensive effect of angiotensin converting enzyme (ACE) inhibitors, angiotensin receptor blockers (ARBs), or beta-blockers (including propranolol).<br>• In patients who are elderly, volume-depleted (including those on diuretic therapy), or have renal impairment, co-administration of an NSAID with ACE inhibitors or ARBs may result in deterioration of renal function, including possible acute renal failure. These effects are usually reversible.
Intervention:	• During concomitant use of Naproxen and ACE-inhibitors, ARBs, or beta-blockers, monitor blood pressure to ensure that the desired blood pressure is obtained.<br>• During concomitant use of Naproxen and ACE-inhibitors or ARBs in patients who are elderly, volume-depleted, or have impaired renal function, monitor for signs of worsening renal function *(see WARNINGS: Renal Toxicity and Hyperkalemia).*<br>• When these drugs are administered concomitantly, patients should be adequately hydrated. Assess renal function at the beginning of the concomitant treatment and periodically thereafter.
**Diuretics**	
Clinical Impact:	Clinical studies, as well as post-marketing observations, showed that NSAIDs reduced the natriuretic effect of loop diuretics (e.g., furosemide) and thiazide diuretics in some patients. This effect has been attributed to the NSAID inhibition of renal prostaglandin synthesis.
Intervention	During concomitant use of Naproxen with diuretics, observe patients for signs of worsening renal function, in addition to assuring diuretic efficacy including antihypertensive effects (see *WARNINGS: Renal Toxicity and Hyperkalemia*).
**Digoxin**	
Clinical Impact:	The concomitant use of Naproxen with digoxin has been reported to increase the serum concentration and prolong the half-life of digoxin.
Intervention:	During concomitant use of Naproxen and digoxin, monitor serum digoxin levels.
**Lithium**	
Clinical Impact:	NSAIDs have produced elevations in plasma lithium levels and reductions in renal lithium clearance. The mean minimum lithium concentration increased 15%, and the renal clearance decreased by approximately 20%. This effect has been attributed to NSAID inhibition of renal prostaglandin synthesis.
Intervention:	During concomitant use of Naproxen and lithium, monitor patients for signs of lithium toxicity.
**Methotrexate**	
Clinical Impact:	Concomitant use of NSAIDs and methotrexate may increase the risk for methotrexate toxicity (e.g., neutropenia, thrombocytopenia, renal dysfunction).
Intervention:	During concomitant use of Naproxen and methotrexate, monitor patients for methotrexate toxicity.
**Cyclosporine**	
Clinical Impact:	Concomitant use of Naproxen and cyclosporine may increase **cyclosporine's** nephrotoxicity.
Intervention:	During concomitant use of Naproxen and cyclosporine, monitor patients for signs of worsening renal function.
**NSAIDs and Salicylates**	

Clinical Impact:	Concomitant use of Naproxen with other NSAIDs or salicylates (e.g., diflunisal, salsalate) increases the risk of GI toxicity, with little or no increase in efficacy ***(see** WARNINGS: Gastrointestinal Bleeding, Ulceration and Perforation**).***
Intervention:	The concomitant use of Naproxen with other NSAIDs or salicylates is not recommended.
**Pemetrexed**	
Clinical Impact:	Concomitant use of Naproxen may increase the risk of pemetrexed-associated myelosuppression, renal, and GI toxicity (see the pemetrexed prescribing information).
Intervention:	During concomitant use of Naproxen and pemetrexed, in patients with renal impairment whose creatinine clearance ranges from 45 to 79 mL/min, monitor for myelosuppression, renal and GI toxicity.<br>NSAIDs with short elimination half-lives (e.g., diclofenac, indomethacin) should be avoided for a period of two days before, the day of, and two days following administration of pemetrexed.<br>In the absence of data regarding potential interaction between pemetrexed and NSAIDs with longer half-lives (e.g., meloxicam, nabumetone), patients taking these NSAIDs should interrupt dosing for at least five days before, the day of, and two days following pemetrexed administration.
**Antacids and Sucralfate**	
Clinical Impact:	Concomitant administration of some antacids (magnesium oxide or aluminum hydroxide) and sucralfate can delay the absorption of Naproxen.
Intervention:	Concomitant administration of antacids such as magnesium oxide or aluminum hydroxide, and sucralfate with Naproxen is not recommended.
**Cholestyramine**	
Clinical Impact:	Concomitant administration of cholestyramine can delay the absorption of Naproxen.
Intervention:	Concomitant administration of cholestyramine with Naproxen is not recommended.
**Probenecid**	
Clinical Impact:	Probenecid given concurrently increases Naproxen anion plasma levels and extends its plasma half-life significantly.
Intervention:	Patients simultaneously receiving Naproxen and probenecid should be observed for adjustment of dose if required.
**Other Albumin-Bound Drugs**	
Clinical Impact:	Naproxen is highly bound to plasma albumin; it thus has a theoretical potential for interaction with other albumin-bound drugs such as coumarin-type anticoagulants, sulphonylureas, hydantoins, other NSAIDs, and aspirin ***(see****WARNINGS: Gastrointestinal Bleeding, Ulceration, and Perforation*).
Intervention:	Patients simultaneously receiving Naproxen and a hydantoin, sulphonamide or sulphonylurea should be observed for adjustment of dose if required.

Image/Content by the Federal Drug Administration, retrieved from: https://www.accessdata.fda.gov/drugsatfda_docs/label/2016/018965s022s023lbl.pdf on August 30, 2017. Image/Content is in the public domain.
