# Supplementary material for: Pharmacology, Pharmacotherapy, and Pharmacopolicy Through an Evidence-Based Medicine: A Novel Approach for First-Year Medical Students
Source: MedEdPORTAL. 2020 Jul 20;16:10934. doi: 10.15766/mep_2374-8265.10934 (PMC7373350; doi:10.15766/mep_2374-8265.10934)
Supplement: Supplementary file 1 — Activity Information.docxUSDA QuickSheet.pdfFDA QuickSheet.pdfAdverse vs Side Effects.docxSeating Chart.pdfAcetaminophen Handout.pdfBeano Handout.docxMevacor Handout.pdfNaproxen Handout.pdfPraluent Handout.pdfXenical Handout.pdfFat-Soluble Vitamins Handout.pdfGroup Quiz.docxQuiz Answers.docx [file mep_2374-8265.10934-s001.zip › J. Praluent Handout.pdf]

HIGHLIGHTS OF PRESCRIBING INFORMATION
These highlights do not include all the information needed to use PRALUENT safely and effectively. See full prescribing information for PRALUENT.

PRALUENT™ (alirocumab) injection, for subcutaneous use
Initial U.S. Approval: 2015

----------------------------INDICATIONS AND USAGE--------------------------
PRALUENT is a PCSK9 (Proprotein Convertase Subtilisin Kexin Type 9) inhibitor antibody indicated as adjunct to diet and maximally tolerated statin therapy for the treatment of adults with heterozygous familial hypercholesterolemia or clinical atherosclerotic cardiovascular disease, who require additional lowering of LDL-cholesterol (LDL-C). (1.1)

Limitations of Use
- The effect of PRALUENT on cardiovascular morbidity and mortality has not been determined. (1.2)

---------------------DOSAGE AND ADMINISTRATION----------------------
The recommended starting dose for PRALUENT is 75 mg administered subcutaneously once every 2 weeks, since the majority of patients achieve sufficient LDL-C reduction with this dosage. If the LDL-C response is inadequate, the dosage may be increased to the maximum dosage of 150 mg administered every 2 weeks. (2.1)

Measure LDL-C levels within 4 to 8 weeks of initiating or titrating PRALUENT, to assess response and adjust the dose, if needed. (2.1)

-------------------DOSAGE FORMS AND STRENGTHS-------------------
- Injection: 75 mg/mL or 150 mg/mL solution in a single-dose pre-filled pen (3)
- Injection: 75 mg/mL or 150 mg/mL solution in a single-dose pre-filled syringe (3)

------------------------------CONTRAINDICATIONS----------------------------
History of a serious hypersensitivity reaction to PRALUENT. (4)

----------------------WARNINGS AND PRECAUTIONS---------------------
- Allergic Reactions: Hypersensitivity reactions (e.g., pruritus, rash, urticaria), including some serious events (e.g., hypersensitivity vasculitis and hypersensitivity reactions requiring hospitalization), have been reported with PRALUENT treatment. If signs or symptoms of serious allergic reactions occur, discontinue treatment with PRALUENT, treat according to the standard of care, and monitor until signs and symptoms resolve. (5.1)

----------------------------ADVERSE REACTIONS----------------------------
The most commonly occurring adverse reactions (≥5% of patients treated with PRALUENT and occurring more frequently than with placebo) are nasopharyngitis, injection site reactions, and influenza. (6.1)

To report SUSPECTED ADVERSE REACTIONS, contact Sanofi at 1-800-633-1610 or FDA at 1-800-FDA-1088 or *www.fda.gov/medwatch*.

See 17 for PATIENT COUNSELING INFORMATION and FDA-approved patient labeling.

Revised: 7/2015

4 CONTRAINDICATIONS

PRALUENT is contraindicated in patients with a history of a serious hypersensitivity reaction to PRALUENT. Reactions have included hypersensitivity vasculitis and hypersensitivity reactions requiring hospitalization. [See Warnings and Precautions (5.1)]

5 WARNINGS AND PRECAUTIONS

5.1 Allergic Reactions

Hypersensitivity reactions (e.g., pruritus, rash, urticaria), including some serious events (e.g., hypersensitivity vasculitis and hypersensitivity reactions requiring hospitalization), have been reported with PRALUENT treatment. If signs or symptoms of serious allergic reactions occur, discontinue treatment with PRALUENT, treat according to the standard of care, and monitor until signs and symptoms resolve [see Contraindications (4)].

6.2 Immunogenicity

As with all therapeutic proteins, there is a potential for immunogenicity with PRALUENT. In a pool of ten placebo- and active-controlled trials, 4.8% of patients treated with PRALUENT had anti-drug antibodies (ADA) newly detected after initiating treatment as compared with 0.6% of patients treated with control.

Patients who developed ADA had a higher incidence of injection site reactions compared with patients who did not develop ADA (10.2% vs 5.9%).

A total of 1.2% of patients treated with PRALUENT developed neutralizing antibodies (NAb) on at least one occasion as compared with no patients treated with control, and 0.3% of patients both tested positive for NAb and exhibited transient or prolonged loss of efficacy. The long-term consequences of continuing PRALUENT treatment in the presence of persistent NAb are unknown.

Immunogenicity data are highly dependent on the sensitivity and specificity of the assay as well as other factors. Additionally, the observed incidence of antibody positivity in an assay may be influenced by several factors, including sample handling, timing of sample collection, concomitant medications, and underlying disease. For these reasons, comparison of the incidence of antibodies to PRALUENT with the incidence of antibodies to other products may be misleading.

PRALUENT is a sterile, preservative-free, clear, colorless to pale yellow solution for subcutaneous injection. PRALUENT 75 mg/mL or 150 mg/mL solution for subcutaneous injection in a single-dose pre-filled pen or single-dose pre-filled syringe is supplied in a siliconized 1 mL Type-1 clear glass syringe. The needle shield is not made with natural rubber latex.

Each 75 mg/mL pre-filled pen or pre-filled syringe contains histidine (8 mM), polysorbate 20 (0.1 mg), sucrose (100 mg), and Water for Injection USP, to pH 6.0.

Each 150 mg/mL pre-filled pen or pre-filled syringe contains histidine (6 mM), polysorbate 20 (0.1 mg), sucrose (100 mg), and Water for Injection USP, to pH 6.0.

12 CLINICAL PHARMACOLOGY

12.1 Mechanism of Action

Alirocumab is a human monoclonal antibody that binds to proprotein convertase subtilisin kexin type 9 (PCSK9). PCSK9 binds to the low-density lipoprotein receptors (LDLR) on the surface of hepatocytes to promote LDLR degradation within the liver. LDLR is the primary receptor that clears circulating LDL, therefore the decrease in LDLR levels by PCSK9 results in higher blood levels of LDL-C. By inhibiting the binding of PCSK9 to LDLR, alirocumab increases the number of LDLRs available to clear LDL, thereby lowering LDL-C levels.

12.2 Pharmacodynamics

Alirocumab reduced free PCSK9 in a concentration-dependent manner. Following a single subcutaneous administration of alirocumab 75 or 150 mg, maximal suppression of free PCSK9 occurred within 4 to 8 hours. Free PCSK9 concentrations returned to baseline when alirocumab concentrations decreased below the limit of quantitation.

Image/Content by the Federal Drug Administration, retrieved from: https://www.accessdata.fda.gov/drugsatfda_docs/label/2018/125559s015lbl.pdf on August 30, 2017. Image/Content is in the public domain.
