# Supplementary material for: Pharmacology, Pharmacotherapy, and Pharmacopolicy Through an Evidence-Based Medicine: A Novel Approach for First-Year Medical Students
Source: MedEdPORTAL. 2020 Jul 20;16:10934. doi: 10.15766/mep_2374-8265.10934 (PMC7373350; doi:10.15766/mep_2374-8265.10934)
Supplement: Supplementary file 1 — Activity Information.docxUSDA QuickSheet.pdfFDA QuickSheet.pdfAdverse vs Side Effects.docxSeating Chart.pdfAcetaminophen Handout.pdfBeano Handout.docxMevacor Handout.pdfNaproxen Handout.pdfPraluent Handout.pdfXenical Handout.pdfFat-Soluble Vitamins Handout.pdfGroup Quiz.docxQuiz Answers.docx [file mep_2374-8265.10934-s001.zip › K. Xenical Handout.pdf]

HIGHLIGHTS OF PRESCRIBING INFORMATION
These highlights do not include all the information needed to use XENICAL safely and effectively. See full prescribing information for XENICAL

XENICAL (orlistat) Capsules for oral use
Initial U.S. Approval: 1999

RECENT MAJOR CHANGES

Warnings and Precautions (5.1) 8/2016

INDICATIONS AND USAGE

- XENICAL is a reversible inhibitor of gastrointestinal lipases indicated for obesity management including weight loss and weight maintenance when used in conjunction with a reduced-calorie diet. (1)
- XENICAL is also indicated to reduce the risk for weight regain after prior weight loss. (1)

DOSAGE AND ADMINISTRATION

- One 120-mg capsule three times a day with each main meal containing fat (during or up to 1 hour after the meal). (2)
- Advise patients to take a nutritionally balanced, reduced-calorie diet that contains approximately 30% of calories from fat. (2)
- Distribute the daily intake of fat, carbohydrate, and protein over three main meals. (2)
- Advise patients to take a multivitamin containing fat-soluble vitamins to ensure adequate nutrition. (2)
- Take the vitamin supplement at least 2 hours before or after the administration of XENICAL, such as at bedtime. (2)
- For patients receiving both XENICAL and cyclosporine therapy, administer cyclosporine 3 hours after XENICAL. (2)
- For patients receiving both XENICAL and levothyroxine therapy, administer levothyroxine and XENICAL at least 4 hours apart. (2)

DOSAGE FORMS AND STRENGTHS

- XENICAL 120 mg Capsules. (3)

CONTRAINDICATIONS

- Pregnancy (4, 8.1)
- Chronic malabsorption syndrome (4)
- Cholestasis (4)
- Known hypersensitivity to XENICAL or to any component of this product (4)

WARNINGS AND PRECAUTIONS

- XENICAL has drug interactions and can decrease vitamin absorption. (5.1,7)
- Take a multivitamin supplement that contains fat-soluble vitamins to ensure adequate nutrition. (5.1)
- Rare cases of severe liver injury with hepatocellular necrosis or acute hepatic failure have been reported. (5.2)
- Patients may develop increased levels of urinary oxalate following treatment with XENICAL. Monitor renal function in patients at risk for renal insufficiency. (5.3)
- Substantial weight loss can increase the risk of cholelithiasis. (5.4)
- Exclude organic causes of obesity (eg, hypothyroidism) before prescribing XENICAL. (5.5)
- Gastrointestinal events may increase when XENICAL is taken with a diet high in fat (>30% total daily calories from fat). (5.5)

ADVERSE REACTIONS

Most common treatment emergent adverse reactions (≥5% and at least twice that of placebo) include oily spotting, flatus with discharge, fecal urgency, fatty/oily stool, oily evacuation, increased defecation and fecal incontinence. (6.1)

To report SUSPECTED ADVERSE REACTIONS, contact Genentech at 1-888-835-2555 or FDA at 1-800-FDA-1088 or www.fda.gov/medwatch.

DRUG INTERACTIONS

- **Cyclosporine:** Reduction in cyclosporine plasma levels was observed when XENICAL was coadministered with cyclosporine. (7.1)
- **Fat-soluble Vitamin Supplements and Analogues:** All patients should take a daily multivitamin that contains vitamins A, D, E, K, and beta-carotene. (7.2)
- **Levothyroxine:** Patients treated concomitantly with XENICAL and levothyroxine should be monitored for changes in thyroid function. (7.3)
- **Warfarin:** Patients on chronic stable doses of warfarin who are prescribed XENICAL should be monitored closely for changes in coagulation parameters. (7.4)
- **Amiodarone:** A reduction in exposure to amiodarone was observed when XENICAL was co-administered. (7.5)
- **Antiepileptic Drugs:** Convulsions have been reported in patients taking XENICAL with antiepileptic drugs. Patients should be monitored for possible changes in frequency or severity of convulsions. (7.6)
- **Antiretroviral Drugs:** Loss of virological control has been reported in HIV-infected patients. Patients should be monitored frequently for changes in HIV RNA levels. (7.7)

USE IN SPECIFIC POPULATIONS

- Nursing Mothers: Caution should be exercised when administered to a nursing woman. (8.3)

See 17 for PATIENT COUNSELING INFORMATION and FDA-approved patient labeling.

Revised: 8/2016

Based on fecal fat measurements, the effect of XENICAL is seen as soon as 24 to 48 hours after dosing. Upon discontinuation of therapy, fecal fat content usually returns to pretreatment levels within 48 to 72 hours.

3 DOSAGE FORMS AND STRENGTHS

XENICAL 120 mg turquoise capsules imprinted with ROCHE and XENICAL 120 in black ink.

4 CONTRAINDICATIONS

XENICAL is contraindicated in:

- Pregnancy *[see Use in Specific Populations (8.1)]*
- Patients with chronic malabsorption syndrome
- Patients with cholestasis
- Patients with known hypersensitivity to XENICAL or to any component of this product

5 WARNINGS AND PRECAUTIONS

5.1 Drug Interactions and Decreased Vitamin Absorption

XENICAL may interact with concomitant drugs including cyclosporine, levothyroxine, warfarin, amiodarone, antiepileptic drugs, and antiretroviral drugs *[see Drug Interactions (7)]*.

Data from a XENICAL and cyclosporine drug interaction study indicate a reduction in cyclosporine plasma levels when XENICAL was coadministered with cyclosporine. Therefore, XENICAL and cyclosporine should not be simultaneously coadministered. To reduce the chance of a drug-drug interaction, cyclosporine should be taken at least 3 hours before or after XENICAL in patients taking both drugs. In addition, in those patients whose cyclosporine levels are being measured, more frequent monitoring should be considered.

Patients should be strongly encouraged to take a multivitamin supplement that contains fat-soluble vitamins to ensure adequate nutrition because XENICAL has been shown to reduce the absorption of some fat-soluble vitamins and beta-carotene *[see Dosage and Administration (2), and Adverse Reactions (6.1)]*. In addition, the levels of vitamin D and beta-carotene may be low in obese patients compared with non-obese subjects. The supplement should be taken once a day at least 2 hours before or after the administration of XENICAL, such as at bedtime.

Weight-loss may affect glycemic control in patients with diabetes mellitus. A reduction in dose of oral hypoglycemic medication (e.g., sulfonylureas) or insulin may be required in some patients *[see Clinical Studies (14)]*.

5.2 Liver Injury

There have been rare postmarketing reports of severe liver injury with hepatocellular necrosis or acute hepatic failure in patients treated with XENICAL, with some of these cases resulting in liver transplant or death. Patients should be instructed to report any symptoms of hepatic dysfunction (anorexia, pruritus, jaundice, dark urine, light-colored stools, or right upper quadrant pain) while taking XENICAL. When these symptoms occur, XENICAL and other suspect medications should be discontinued immediately and liver function tests and ALT and AST levels obtained.

5.3 Increases in Urinary Oxalate

Some patients may develop increased levels of urinary oxalate following treatment with XENICAL. Cases of oxalate nephrolithiasis and oxalate nephropathy with renal failure have been reported. Monitor renal function when prescribing XENICAL to patients at risk for renal impairment and use with caution in those with a history of hyperoxaluria or calcium oxalate nephrolithiasis.

5.4 Cholelithiasis

Substantial weight loss can increase the risk of cholelithiasis. In a clinical trial of XENICAL for the prevention of type 2 diabetes, the rates of cholelithiasis as an adverse event were 2.9% (47/1649) for patients randomized to XENICAL and 1.8% (30/1655) for patients randomized to placebo.

5.5 Miscellaneous

Organic causes of obesity (e.g., hypothyroidism) should be excluded before prescribing XENICAL.

Patients should be advised to adhere to dietary guidelines *[see Dosage and Administration (2)]*. Gastrointestinal events *[see Adverse Reactions (6.1)]* may increase when XENICAL is taken with a diet high in fat (>30% total daily calories from fat). The daily intake of fat should be distributed over three main meals. If XENICAL is taken with any one meal very high in fat, the possibility of gastrointestinal effects increases.

6 ADVERSE REACTIONS

6.1 Clinical Trials

Because clinical trials are conducted under widely varying conditions, adverse reaction rates observed in the clinical trials of a drug cannot be directly compared to rates in the clinical trials of another drug and may not reflect the rates observed in patients.

Commonly Observed (based on first year and second year data)

Gastrointestinal (GI) symptoms were the most commonly observed treatment-emergent adverse events associated with the use of XENICAL in the seven double-blind, placebo-controlled clinical trials and are primarily a manifestation of the mechanism of action. (Commonly observed is defined as an incidence of ≥5% and an incidence in the XENICAL 120 mg group that is at least twice that of placebo.)

Table 2 Commonly Observed Adverse Events

	Year 1		Year 2	
**Adverse Event**	**XENICAL* % Patients (N=1913)**	**Placebo* % Patients (N=1466)**	**XENICAL* % Patients (N=613)**	**Placebo* % Patients (N=524)**
Oily Spotting†	26.6	1.3	4.4	0.2
Flatus with Discharge	23.9	1.4	2.1	0.2
Fecal Urgency	22.1	6.7	2.8	1.7
Fatty/Oily Stool†	20.0	2.9	5.5	0.6
Oily Evacuation†	11.9	0.8	2.3	0.2
Increased Defecation	10.8	4.1	2.6	0.8
Fecal Incontinence	7.7	0.9	1.8	0.2

*Treatment designates XENICAL three times a day plus diet or placebo plus diet
†Oily discharge may be clear or have a coloration such as orange or brown.

In general, the first occurrence of these events was within 3 months of starting therapy. Overall, approximately 50% of all episodes of GI adverse events associated with XENICAL treatment lasted for less than 1 week, and a majority lasted for no more than 4 weeks. However, GI adverse events may occur in some individuals over a period of 6 months or longer.

6.2 Postmarketing Experience

The following adverse reactions have been identified during postapproval use of XENICAL. Because these reactions are reported voluntarily from a population of uncertain size, it is not always possible to reliably estimate their frequency or establish a causal relationship to XENICAL exposure.

- Rare cases of increase in transaminases and in alkaline phosphatase and hepatitis that may be serious have been reported. There have been reports of hepatic failure observed with the use of XENICAL in postmarketing surveillance, with some of these cases resulting in liver transplant or death *[see Warnings and Precautions (5.2)]*.
- Rare cases of hypersensitivity have been reported with the use of XENICAL. Signs and symptoms have included pruritus, rash, urticaria, angioedema, bronchospasm and anaphylaxis. Very rare cases of bullous eruption have been reported.

- Rare cases of leukocytoclastic vasculitis have been reported. Clinical signs include palpable purpura, maculopapular lesions, or bullous eruption.
- Acute oxalate nephropathy after treatment with XENICAL has been reported in patients with or at risk for renal disease [see Warnings and Precautions (5.3)].
- Pancreatitis has been reported with the use of XENICAL in postmarketing surveillance. No causal relationship or physiopathological mechanism between pancreatitis and obesity therapy has been definitively established.
- Lower gastrointestinal bleeding has been reported in patients treated with XENICAL. Most reports are nonserious; severe or persistent cases should be investigated further.

7 DRUG INTERACTIONS

7.1 Cyclosporine

Data from a XENICAL and cyclosporine drug interaction study indicate a reduction in cyclosporine plasma levels when XENICAL was coadministered with cyclosporine. XENICAL and cyclosporine should not be simultaneously coadministered. Cyclosporine should be administered 3 hours after the administration of XENICAL [see Dosage and Administration (2), and Warnings and Precautions (5.1)].

7.2 Fat-soluble Vitamin Supplements and Analogues

Data from a pharmacokinetic interaction study showed that the absorption of beta-carotene supplement is reduced when concomitantly administered with XENICAL. XENICAL inhibited absorption of a vitamin E acetate supplement. The effect of XENICAL on the absorption of supplemental vitamin D, vitamin A, and nutritionally-derived vitamin K is not known at this time [see Clinical Pharmacology (12.3), and Warnings and Precautions (5.1)].

7.3 Levothyroxine

Hypothyroidism has been reported in patients treated concomitantly with XENICAL and levothyroxine postmarketing. Patients treated concomitantly with XENICAL and levothyroxine should be monitored for changes in thyroid function. Administer levothyroxine and XENICAL at least 4 hours apart [see Dosage and Administration (2)].

7.4 Anticoagulants including Warfarin

Vitamin K absorption may be decreased with XENICAL. Reports of decreased prothrombin, increased INR and unbalanced anticoagulant treatment resulting in change of hemostatic parameters have been reported in patients treated concomitantly with XENICAL and anticoagulants. Patients on chronic stable doses of warfarin or other anticoagulants who are prescribed XENICAL should be monitored closely for changes in coagulation parameters [see Clinical Pharmacology (12.3)].

7.5 Amiodarone

A pharmacokinetic study, where amiodarone was orally administered during orlistat treatment, demonstrated a reduction in exposure to amiodarone and its metabolite, desethylamiodarone [see Clinical Pharmocology (12.3)]. A reduced therapeutic effect of amiodarone is possible. The effect of commencing orlistat treatment in patients on stable amiodarone therapy has not been studied.

7.6 Antiepileptic Drugs

Convulsions have been reported in patients treated concomitantly with orlistat and antiepileptic drugs. Patients should be monitored for possible changes in the frequency and/or severity of convulsions.

7.7 Antiretroviral Drugs

Loss of virological control has been reported in HIV-infected patients taking orlistat concomitantly with antiretroviral drugs such as atazanavir, ritonavir, tenofovir disoproxil fumarate, emtricitabine, and with the combinations lopinavir/ritonavir and emtricitabine/efavirenz/tenofovir disoproxil fumarate. The exact mechanism for this is unclear, but may include a drug-drug interaction that inhibits systemic absorption of the antiretroviral drug. HIV RNA levels should be frequently monitored in patients who take XENICAL while being treated for HIV infection. If there is a confirmed increase in HIV viral load, XENICAL should be discontinued.

8 USE IN SPECIFIC POPULATIONS

8.1 Pregnancy

Pregnancy Category X
XENICAL is contraindicated during pregnancy, because weight loss offers no potential benefit to a pregnant woman and may result in fetal harm. A minimum weight gain, and no weight loss, is currently recommended for all pregnant women, including those who are already overweight or obese, due to the obligatory weight gain that occurs in maternal tissues during pregnancy. No embryotoxicity or teratogenicity was seen in animals that received orlistat at doses much higher than the recommended human dose. If this drug is used during pregnancy, or if the patient becomes pregnant while taking this drug, the patient should be apprised of the potential hazard of maternal weight loss to the fetus.

12.1 Mechanism of Action

Orlistat is a reversible inhibitor of gastrointestinal lipases. It exerts its therapeutic activity in the lumen of the stomach and small intestine by forming a covalent bond with the active serine residue site of gastric and pancreatic lipases. The inactivated enzymes are thus unavailable to hydrolyze dietary fat in the form of triglycerides into absorbable free fatty acids and monoglycerides. As undigested triglycerides are not absorbed, the resulting caloric deficit may have a positive effect on weight control.

Additional info for SIG 3 - Fat soluble vitamins

The fat-soluble vitamins A, D, E and K, are absorbed in the intestine in the presence of fat. Classical deficiencies of these vitamins can manifest clinically as night blindness (vitamin A), osteomalacia (vitamin D), increased oxidative cell stress (vitamin E) and haemorrhage (vitamin K).

Vitamin A is involved in immune function, vision, reproduction, and cellular communication. Vitamin A is critical for vision as an essential component of rhodopsin, a protein that absorbs light in the retinal receptors, and because it supports the normal differentiation and functioning of the conjunctival membranes and cornea. Vitamin A also supports cell growth and differentiation,

Two forms of vitamin A are available in the human diet: preformed vitamin A (retinol and its esterified form, retinyl ester) and provitamin A carotenoids. Preformed vitamin A is found in foods from animal sources, including dairy products, fish, and meat (especially liver). the most important provitamin A carotenoid is beta-carotene. Both provitamin A and preformed vitamin A must be metabolized intracellularly to retinal and retinoic acid, the active forms of vitamin A, to support the vitamin's important biological functions.

Vitamin D is a fat-soluble vitamin that is naturally present in very few foods, added to others, and available as a dietary supplement. It is also produced endogenously when ultraviolet rays from sunlight strike the skin and trigger vitamin D synthesis. Vitamin D promotes calcium absorption in the gut and maintains adequate serum calcium and phosphate concentrations to enable normal mineralization of bone and to prevent hypocalcemic tetany. It is also needed for bone growth and bone remodeling by osteoblasts and osteoclasts. Without sufficient vitamin D, bones can become thin, brittle, or misshapen. Vitamin D sufficiency prevents rickets in children and osteomalacia in adults. Together with calcium, vitamin D also helps protect older adults from osteoporosis. Vitamin D has other roles in the body, including modulation of cell growth, neuromuscular and immune function, and reduction of inflammation

Vitamin E is the collective name for a group of fat-soluble compounds with distinctive antioxidant activities. Vitamin E is important for the normal morphology of erythrocytes and is thought to be involved in slowing the aging process, since it is essential for the elimination of reactive oxygen species (ROS), which are involved in cell destruction.[58] Furthermore, this vitamin inhibits platelet aggregations, and therefore it may play a protective role against the atherosclerotic process and cardiovascular disease. It has also been suggested that vitamin E has a protective role against arthritis, cataracts, neurological disease and immunological disorders.

Deficiency symptoms include peripheral neuropathy, ataxia, skeletal myopathy, retinopathy, and impairment of the immune response. People with Crohn's disease, cystic fibrosis, or an inability to secrete bile from the liver into the digestive tract, for example, often pass greasy stools or have chronic diarrhea; as a result, they sometimes require water-soluble forms of vitamin E.

Vitamin K is a group of structurally similar, fat-soluble vitamins the human body requires for complete synthesis of certain proteins that are prerequisites for blood coagulation and which the body also needs for controlling binding of calcium in bones and other tissues. The vitamin K-related modification of the proteins allows them to bind calcium ions, which they cannot do otherwise. Without vitamin K, blood coagulation is seriously impaired, and uncontrolled bleeding occurs.

Image/Content by the Federal Drug Administration, retrieved from: https://www.accessdata.fda.gov/drugsatfda_docs/label/2012/020766s029lbl.pdf on August 30, 2017. Image/Content is in the public domain.
