# Supplementary material for: Pharmacology, Pharmacotherapy, and Pharmacopolicy Through an Evidence-Based Medicine: A Novel Approach for First-Year Medical Students
Source: MedEdPORTAL. 2020 Jul 20;16:10934. doi: 10.15766/mep_2374-8265.10934 (PMC7373350; doi:10.15766/mep_2374-8265.10934)
Supplement: Supplementary file 1 — Activity Information.docxUSDA QuickSheet.pdfFDA QuickSheet.pdfAdverse vs Side Effects.docxSeating Chart.pdfAcetaminophen Handout.pdfBeano Handout.docxMevacor Handout.pdfNaproxen Handout.pdfPraluent Handout.pdfXenical Handout.pdfFat-Soluble Vitamins Handout.pdfGroup Quiz.docxQuiz Answers.docx [file mep_2374-8265.10934-s001.zip › L. Fat-Soluble Vitamins Handout.pdf]

The fat-soluble vitamins A, D, E and K, are absorbed in the intestine in the presence of fat. Classical deficiencies of these vitamins can manifest clinically as night blindness (vitamin A), osteomalacia (vitamin D), increased oxidative cell stress (vitamin E) and haemorrhage (vitamin K).

Vitamin A is involved in immune function, vision, reproduction, and cellular communication. Vitamin A is critical for vision as an essential component of rhodopsin, a protein that absorbs light in the retinal receptors, and because it supports the normal differentiation and functioning of the conjunctival membranes and cornea. Vitamin A also supports cell growth and differentiation,

Two forms of vitamin A are available in the human diet: preformed vitamin A (retinol and its esterified form, retinyl ester) and provitamin A carotenoids. Preformed vitamin A is found in foods from animal sources, including dairy products, fish, and meat (especially liver). the most important provitamin A carotenoid is beta-carotene. Both provitamin A and preformed vitamin A must be metabolized intracellularly to retinal and retinoic acid, the active forms of vitamin A, to support the vitamin's important biological functions.

Vitamin D is a fat-soluble vitamin that is naturally present in very few foods, added to others, and available as a dietary supplement. It is also produced endogenously when ultraviolet rays from sunlight strike the skin and trigger vitamin D synthesis. Vitamin D promotes calcium absorption in the gut and maintains adequate serum calcium and phosphate concentrations to enable normal mineralization of bone and to prevent hypocalcemic tetany. It is also needed for bone growth and bone remodeling by osteoblasts and osteoclasts. Without sufficient vitamin D, bones can become thin, brittle, or misshapen. Vitamin D sufficiency prevents rickets in children and osteomalacia in adults. Together with calcium, vitamin D also helps protect older adults from osteoporosis. Vitamin D has other roles in the body, including modulation of cell growth, neuromuscular and immune function, and reduction of inflammation

Vitamin E is the collective name for a group of fat-soluble compounds with distinctive antioxidant activities. Vitamin E is important for the normal morphology of erythrocytes and is thought to be involved in slowing the aging process, since it is essential for the elimination of reactive oxygen species (ROS), which are involved in cell destruction.[58] Furthermore, this vitamin inhibits platelet aggregations, and therefore it may play a protective role against the atherosclerotic process and cardiovascular disease. It has also been suggested that vitamin E has a protective role against arthritis, cataracts, neurological disease and immunological disorders.

Deficiency symptoms include peripheral neuropathy, ataxia, skeletal myopathy, retinopathy, and impairment of the immune response. People with Crohn's disease, cystic fibrosis, or an inability to secrete bile from the liver into the digestive tract, for example, often pass greasy stools or have chronic diarrhea; as a result, they sometimes require water-soluble forms of vitamin E.

Vitamin K is a group of structurally similar, fat-soluble vitamins the human body requires for complete synthesis of certain proteins that are prerequisites for blood coagulation and which the body also needs for controlling binding of calcium in bones and other tissues. The vitamin K-related modification of the proteins allows them to bind calcium ions, which they cannot do otherwise. Without vitamin K, blood coagulation is seriously impaired, and uncontrolled bleeding occurs.

Image/Content by the National Institutes for Health, retrieved from: https://ods.od.nih.gov/factsheets/vitamina-Health%20Professional/, https://ods.od.nih.gov/factsheets/VitaminD-HealthProfessional/, https://ods.od.nih.gov/factsheets/VitaminE-HealthProfessional/ on August 30, 2017. Image/Content is in the public domain.
