# Supplementary material for: Pharmacology, Pharmacotherapy, and Pharmacopolicy Through an Evidence-Based Medicine: A Novel Approach for First-Year Medical Students
Source: MedEdPORTAL. 2020 Jul 20;16:10934. doi: 10.15766/mep_2374-8265.10934 (PMC7373350; doi:10.15766/mep_2374-8265.10934)
Supplement: Supplementary file 1 — Activity Information.docxUSDA QuickSheet.pdfFDA QuickSheet.pdfAdverse vs Side Effects.docxSeating Chart.pdfAcetaminophen Handout.pdfBeano Handout.docxMevacor Handout.pdfNaproxen Handout.pdfPraluent Handout.pdfXenical Handout.pdfFat-Soluble Vitamins Handout.pdfGroup Quiz.docxQuiz Answers.docx [file mep_2374-8265.10934-s001.zip › M. Group Quiz.docx]

**SIG 3 QUIZ**

**Please use the following information to answer both Questions 1 and 2.**

A study is looking at the use of new investigational drug X in patients with metastatic colorectal cancer. Using a multi-center enrollment protocol, the investigators screened 3472 patients for inclusion which subsequently lead to randomization of 3212 patients to receive drug X (n=2409) or placebo (n=803). The primary endpoint is overall survival in months.

1. **Based on the information provided, this study is most likely classified as which of the following?**
2. Phase 1
3. Phase 2
4. Phase 3
5. Phase 4
6. Cannot be determined
7. **The approval of drug X would be determined by which one of the following agencies?**
8. CDC
9. NIH
10. HHS
11. USDA
12. FDA
13. **Which of the following is not a component of the Investigational New Drug (IND) application?**
14. Drug composition
15. Manufacturing procedures
16. Animal toxicology data
17. Human safety data
18. Clinical study designs
19. **MW is a 32-year-old male patient that presents to the clinic complaining of muscle pain for two weeks. While performing a history you note that he had an allergic reaction from naproxen, a non-steroidal anti-inflammatory drug (NSAID), which resulted in his “throat closing and he needed to use his Epi-pen”. You present the patient to the attending and recommend that MW gets a prescription for ibuprofen (NSAID) for his back pain. The attending states that NSAIDs typically have a high rate of cross-reactivity in regards to allergies, so giving ibuprofen would be inappropriate. This principle is an example of which of the following?**
20. Relative contraindication
21. Absolute contraindication
22. Dose-related adverse drug reaction
23. Side-effect
24. Idiosyncratic adverse drug reaction
25. **In 2016, the patent for AstraZenca’s drug Crestor (rosuvastatin) expired. For a company to produce a generic alternative to Crestor, they must file which of the following?**
26. NDA
27. IND
28. ANDA
29. USDA
30. SNDA
31. **Orlistat is available as a prescription drug called Xenical, as well as an OTC called Alli. What are the differences between these two products?**
    1. The name only
    2. The frequency of administration
    3. The side effects
    4. The dosage
32. **Which one of the following statements is true?**
33. Xenical irreversibly inhibits gastric and pancreatic lipases, thereby inhibiting fat absorption
34. Xenical decreases fecal excretion, flatulence
35. Xenical and cyclosporin are contraindicated and must not be prescribed at the same time.
36. Xenical may increase the absorption of vitamins A, D, E, and K.
37. Xenical administered to patients is primarily eliminated in feces
38. **Which finding for a patient who has been taking Xenical (orlistat) is most important to report to the health care provider?**
39. The patient has a chronic cough
40. The patient frequently has liquid stools
41. The patient complains of bloating after meals
42. The patient is experiencing a weight loss plateau
43. The patient is pale and has many bruises
44. **One of your patients JD has been taking Xenical (orlistat) for the past year, and she has recently found out she is pregnant. Which one of the following statements is true:**
    1. Xenical is contraindicated during pregnancy
    2. Xenical is minimally absorbed, thus there is little concern
    3. Xenical requires monitoring of fetal development for proper weight gain
    4. Xenical should be replaced by the OTC Alli
45. **Aspirin can cause problems with which of these conditions?**
46. Bronchitis
47. Sinusitis
48. Asthma
49. Low blood pressure
50. **People allergic to aspirin may also be allergic to which other OTC(s)?**
51. Naproxen
52. Ketoprofen
53. indomethacin
54. Ibuprofen
55. All of the above
56. **Acetaminophen is less likely than aspirin to cause gastric distress, but an overdose of as little as 4 grams of acetaminophen can lead to irreversible liver disease. Who is most at risk?**
57. Smokers
58. Anyone on a diet
59. People who drink alcohol
60. A and C
61. **Which of the following is the most common adverse effect of statin medications?**
62. Rhabdomyolitis
63. Renal failure
64. Liver dysfunction
65. Encephalopathy
66. Hyperkalemia
67. **Beano is an OTC dietary supplement for reducing flatulence and bloating. The molecular mechanism of Beano is which one of the following?**
68. Increased intestinal mobility
69. Decreased intestinal mobility
70. Alpha-glucosidase
71. Alpha-glucolipase
72. Alpha-galactosidase
73. **A patient presents with one of the following conditions. Which one of these can be treated effectively with Beano?**
74. FLOTUS
75. FLATUS
76. POTUS
77. BLOATUS
78. SCOTUS
79. **You’re a third-year medical student on rounds and you’re seeing TS, a 54 year-old female patient that has just been diagnosed with primary hyperlipidemia. As a good medical student you paid attention in your MCS lectures and decide to start her on Praulent (alirocumab) 75mg SC every two weeks. The attending asks you how you plan to monitor her therapy. You reply:**
80. Blood sugar checks three times a day with meals
81. Triglyceride levels in 8 weeks
82. LDL-C levels in 4 weeks
83. HDL levels in 6 weeks
84. Chylomicron levels in 8 weeks
85. **RB is 47-year-old male who is a new patient to your office because his previous doctor no longer accepts his insurance. During the history RB reports he has “high blood fats” and was prescribed a medication, first at a low dose that did not help his problem, which was then increased to a higher dose that “made his muscles hurt a lot” and his “pee turned reddish brown”. He no longer takes that medication, but he cannot remember the name. He says he religiously eats a healthy low fat diet and excercises, but his lab results show severe hyperlipidemia. Which of the following do you consider to be the most likely best option for RB?**
86. Aspirin (NSAID)
87. Alirocumab
88. Atorvastatin
89. Alli (lipase inhibitor)
90. Alpha-galactosidase inhibitor
91. **You are shadowing at a family clinic in Newark, and your preceptor asks you to elicit a history from a 60-year old man who just recently came to the clinic. You take a good history, and determine that his previous doctor diagnosed him with high cholesterol, prescribed Lipitor, and explained options for his lifestyle modifications. The patient, however, insisted he had a special cultural diet that included grapefruit juice, and thus would not be able to adjust his lifestyle to accommodate for that medication. Your preceptor asks you to take the patient’s perspective into account and asks you which drug would NOT be appropriate to manage his high cholesterol, as there is an increased risk of adverse events. You correctly respond:**
92. Mevacor
93. Zetia
94. Niaspan (niacin)
95. Gemfibrozil (fibrate)
96. **As part of your volunteering experience at a fertility clinic, you are supposed to input information from the past medical history of each patient into the electronic record system. You are inputting information for AW, a 30-year old female who is currently pregnant. Her past medical history and current health are significant for high cholesterol, hypertension, and anxiety. TRUE or FALSE: You can start atorvastatin for the management of her high cholesterol at this time.**
97. True
98. False
