# Supplementary material for: Pharmacology, Pharmacotherapy, and Pharmacopolicy Through an Evidence-Based Medicine: A Novel Approach for First-Year Medical Students
Source: MedEdPORTAL. 2020 Jul 20;16:10934. doi: 10.15766/mep_2374-8265.10934 (PMC7373350; doi:10.15766/mep_2374-8265.10934)
Supplement: Supplementary file 1 — Activity Information.docxUSDA QuickSheet.pdfFDA QuickSheet.pdfAdverse vs Side Effects.docxSeating Chart.pdfAcetaminophen Handout.pdfBeano Handout.docxMevacor Handout.pdfNaproxen Handout.pdfPraluent Handout.pdfXenical Handout.pdfFat-Soluble Vitamins Handout.pdfGroup Quiz.docxQuiz Answers.docx [file mep_2374-8265.10934-s001.zip › N. Quiz Answers.docx]

**SIG 3 QUIZ**

**Please use the following information to answer both Questions 1 and 2.**

A study is looking at the use of new investigational drug X in patients with metastatic colorectal cancer. Using a multi-center enrollment protocol, the investigators screened 3472 patients for inclusion which subsequently lead to randomization of 3212 patients to receive drug X (n=2409) or placebo (n=803). The primary endpoint is overall survival in months.

1. **Based on the information provided, this study is most likely classified as which of the following?**
2. Phase 1
3. Phase 2
4. Phase 3
5. Phase 4
6. Cannot be determined

*Correct answer: C*. Based on the number of patients (in the thousands) and the primary endpoint being efficacy, this most closely represents a phase 3 trial. Phase 1 would be much smaller and involve healthy patients (an important exception to this rule is if the drug has significant toxicities such as chemotherapeutic agents). This could be a Phase 2 study, however the number of subjects would be closer to the hundreds. Phase 4 studies only take place once a drug has reached the market. (see FDA poster)

1. **The approval of drug X would be determined by which one of the following agencies?**
2. CDC
3. NIH
4. HHS
5. USDA
6. FDA

*Correct answer: E*. Drug X is a new chemical entity being used to treat metastatic colorectal cancer. Its safety and efficacy data would be reviewed by the FDA once the innovator company submits a completed NDA application.

1. **Which of the following is not a component of the Investigational New Drug (IND) application?**
2. Drug composition
3. Manufacturing procedures
4. Animal toxicology data
5. Human safety data
6. Clinical study designs

*Correct answer: D*. An IND or Investigational New Drug application occurs prior to a company conducting any Phase 1 trials in humans. This must include a variety of data. However it is important to note that an IND cannot contain any human safety or efficacy data, since it must be submitted prior to human trials beginning. Interestingly, there is no actual approval process for IND applications. Currently, if a company submits an IND and is not prohibited from continuing within 30 days, they may commence their Phase 1 trials.

1. **MW is a 32-year-old male patient that presents to the clinic complaining of muscle pain for two weeks. While performing a history you note that he had an allergic reaction from naproxen, a non-steroidal anti-inflammatory drug (NSAID), which resulted in his “throat closing and he needed to use his Epi-pen”. You present the patient to the attending and recommend that MW gets a prescription for ibuprofen (NSAID) for his back pain. The attending states that NSAIDs typically have a high rate of cross-reactivity in regards to allergies, so giving ibuprofen would be inappropriate. This principle is an example of which of the following?**
2. Relative contraindication
3. Absolute contraindication
4. Dose-related adverse drug reaction
5. Side-effect
6. Idiosyncratic adverse drug reaction

*Correct answer: B*. Allergic reactions can present with a wide ranging of severities, the worse being anaphylaxis, or a closure of the airway. While an allergy may occur to just one drug within a class, it is more common for an allergy to be against an entire class of drugs. In this patient he reports an anaphylactic reaction to an NSAID, thus it would be inappropriate to re-challenge him with another NSAID. This case demonstrates the concept of absolute contraindication, or a situation in which a drug may cause a life-threatening event and therefore should not be used.

1. **In 2016, the patent for AstraZenca’s drug Crestor (rosuvastatin) expired. For a company to produce a generic alternative to Crestor, they must file which of the following?**
2. NDA
3. IND
4. ANDA
5. USDA
6. SNDA

*Correct answer:C*. Generic drugs are given an abbreviated approval process since the innovator product has already demonstrate safety and efficacy for that chemical moiety. (see Backgournd material). INDs are filed for a chemical entity prior to human testing (see FDA poster). NDAs are filed for the innovator product to gain FDA approval (see FDA poster). USDA is a branch of the federal government. SNDA is a Supplementary NDA when a company wants to add minor revisions to their NDA, such as changing the label or making a new dosage strength.

1. **Orlistat is available as a prescription drug called Xenical, as well as an OTC called Alli. What are the differences between these two products?**
   1. The name only
   2. The frequency of administration
   3. The side effects
   4. The dosage

*Correct answer: D. Lexicomp search shows that:*

**Dosing: Adult Obesity management:** Oral:

Xenical: 120 mg 3 times daily with each main meal containing fat (during or up to 1 hour after the meal); omit dose if meal is occasionally missed or contains no fat.

Alli: OTC labeling: 60 mg 3 times daily with each main meal containing fat (maximum dose: 180 mg/day).

1. **Which one of the following statements is true?**
2. Xenical irreversibly inhibits gastric and pancreatic lipases, thereby inhibiting fat absorption
3. Xenical decreases fecal excretion, flatulence
4. Xenical and cyclosporin are contraindicated and must not be prescribed at the same time.
5. Xenical may increase the absorption of vitamins A, D, E, and K.
6. Xenical administered to patients is primarily eliminated in feces

*Correct answer:* E. Xenical, also known as orlistat, is administered orally and works by remaining in the gastrointestinal tract in order to curtail fat absorption as an anti-obesity measure.

1. **Which finding for a patient who has been taking Xenical (orlistat) is most important to report to the health care provider?**
2. The patient has a chronic cough
3. The patient frequently has liquid stools
4. The patient complains of bloating after meals
5. The patient is experiencing a weight loss plateau
6. The patient is pale and has many bruises

*Correct answer:* E. Because orlistat blocks the absorption of fat-soluble vitamins, the patient may not be receiving an adequate amount of vitamin K, resulting in a decrease in clotting factors. Abdominal bloating and liquid stools are common side effects of orlistat and indicate that the nurse should remind the patient that fat in the diet may increase these side effects. Weight loss plateaus are normal during weight reduction.

1. **One of your patients JD has been taking Xenical (orlistat) for the past year, and she has recently found out she is pregnant. Which one of the following statements is true:**
   1. Xenical is contraindicated during pregnancy
   2. Xenical is minimally absorbed, thus there is little concern
   3. Xenical requires monitoring of fetal development for proper weight gain
   4. Xenical should be replaced by the OTC Alli

Correct answer is A

Both Lexicomp and Xenical product insert (provided to students) show that Xenical is contraindicated during pregnancy

1. **Aspirin can cause problems with which of these conditions?**
2. Bronchitis
3. Sinusitis
4. Asthma
5. Low blood pressure

Correct answer is C. Aspirin can bring on severe asthma attacks in sensitive individuals.

1. **People allergic to aspirin may also be allergic to which other OTC(s)?**
2. Naproxen
3. Ketoprofen
4. indomethacin
5. Ibuprofen
6. All of the above

*Correct answer is E.* Allergic reactions may include rash, itching and breathing problems. As stated earlier, while an allergy may occur to just one drug within a class, it is more common for an allergy to be against an entire class of drugs.

1. **Acetaminophen is less likely than aspirin to cause gastric distress, but an overdose of as little as 4 grams of acetaminophen can lead to irreversible liver disease. Who is most at risk?**
2. Smokers
3. Anyone on a diet
4. People who drink alcohol
5. A and C

Correct answer is C. Overdosing of acetaminophen alone are toxic to the liver. Alcohol in combination with recommended dosages of acetaminophen increase the risk of liver toxicity.

1. **Which of the following is the most common adverse effect of statin medications?**
2. Rhabdomyolitis
3. Renal failure
4. Liver dysfunction
5. Encephalopathy
6. Hyperkalemia

Correct answer is C. At least 1% of patients taking statins will develop elevated transaminases to the level where the medication must be discontinued. Myositis, elevation of CPK levels or rhabdomyolysis occurs less frequently (<0.1%). Thus, all patients on statins should have their aspartate aminotransferase (**AST**) and alanine aminotransferase (**ALT**), tested routinely to detect liver damage or disorders even in the absence of symptoms. Whereas there is no recommendation to routinely test all patients for creatine phosphokinase (CPK) levels, as an indicator of muscle damage, in the absence of symptoms.

1. **Beano is an OTC dietary supplement for reducing flatulence and bloating. The molecular mechanism of Beano is which one of the following?**
2. Increased intestinal mobility
3. Decreased intestinal mobility
4. Alpha-glucosidase
5. Alpha-glucolipase
6. Alpha-galactosidase

Correct answer is E.

1. **A patient presents with one of the following conditions. Which one of these can be treated effectively with Beano?**
2. FLOTUS
3. FLATUS
4. POTUS
5. BLOATUS
6. SCOTUS

Correct answer is B.

1. **You’re a third-year medical student on rounds and you’re seeing TS, a 54 year-old female patient that has just been diagnosed with primary hyperlipidemia. As a good medical student you paid attention in your MCS lectures and decide to start her on Praulent (alirocumab) 75mg SC every two weeks. The attending asks you how you plan to monitor her therapy. You reply:**
2. Blood sugar checks three times a day with meals
3. Triglyceride levels in 8 weeks
4. LDL-C levels in 4 weeks
5. HDL levels in 6 weeks
6. Chylomicron levels in 8 weeks

*Correct answer: C*. Alirocumab is an injectable drug for the treatment of primary hyperlipidemia. As per Lexicomp and its package insert, therapy should be monitored by “measuring LDL-C levels within 4 to 8 weeks of initiating or titrating PRALUENT, to assess response and adjust the dose, if needed”.

1. **RB is 47-year-old male who is a new patient to your office because his previous doctor no longer accepts his insurance. During the history RB reports he has “high blood fats” and was prescribed a medication, first at a low dose that did not help his problem, which was then increased to a higher dose that “made his muscles hurt a lot” and his “pee turned reddish brown”. He no longer takes that medication, but he cannot remember the name. He says he religiously eats a healthy low fat diet and excercises, but his lab results show severe hyperlipidemia. Which of the following do you consider to be the most likely best option for RB?**
2. Aspirin (NSAID)
3. Alirocumab
4. Atorvastatin
5. Alli (lipase inhibitor)
6. Alpha-galactosidase inhibitor

*Correct answer: B*. The only drug options on this list that treat hyperlipidemia are B and C. This patient is reporting signs and symptoms of rhabdomyolysis, a serious adverse reaction associated with statin use. Rhabdomyolysis is a rapid breakdown of skeletal muscle tissue, which results in an enormous release of electrolytes and myoglobin that can subsequently damage the kidney tubules. Once myoglobin enters the urine, it imparts a reddish-brown color. Given this information, the use of a statin would not be recommended for this patient, thus a PCSK9 inhibitor would be the best option.

1. **You are shadowing at a family clinic in Newark, and your preceptor asks you to elicit a history from a 60-year old man who just recently came to the clinic. You take a good history, and determine that his previous doctor diagnosed him with high cholesterol, prescribed Lipitor, and explained options for his lifestyle modifications. The patient, however, insisted he had a special cultural diet that included grapefruit juice, and thus would not be able to adjust his lifestyle to accommodate for that medication. Your preceptor asks you to take the patient’s perspective into account and asks you which drug would NOT be appropriate to manage his high cholesterol, as there is an increased risk of adverse events. You correctly respond:**
2. Mevacor
3. Zetia
4. Niaspan (niacin)
5. Gemfibrozil (fibrate)

Correct answer: A. This patient expresses the need to continue to eat grapefruits. Grapefruit shows an interaction with all statins that can severely increase serum levels. This information can be found in the package insert or Lexicomp.

1. **As part of your volunteering experience at a fertility clinic, you are supposed to input information from the past medical history of each patient into the electronic record system. You are inputting information for AW, a 30-year old female who is currently pregnant. Her past medical history and current health are significant for high cholesterol, hypertension, and anxiety. TRUE or FALSE: You can start atorvastatin for the management of her high cholesterol at this time.**
2. True
3. False

Correct answer: False. Statins are absolutely contraindicated in pregnancy. They are considered Pregnancy Category X and are NOT to be used under any circumstances in pregnant patients.
