# Supplementary material for: Pharmacology, Pharmacotherapy, and Pharmacopolicy Through an Evidence-Based Medicine: A Novel Approach for First-Year Medical Students
Source: MedEdPORTAL. 2020 Jul 20;16:10934. doi: 10.15766/mep_2374-8265.10934 (PMC7373350; doi:10.15766/mep_2374-8265.10934)
Supplement: Supplementary file 1 — Activity Information.docxUSDA QuickSheet.pdfFDA QuickSheet.pdfAdverse vs Side Effects.docxSeating Chart.pdfAcetaminophen Handout.pdfBeano Handout.docxMevacor Handout.pdfNaproxen Handout.pdfPraluent Handout.pdfXenical Handout.pdfFat-Soluble Vitamins Handout.pdfGroup Quiz.docxQuiz Answers.docx [file mep_2374-8265.10934-s001.zip › _Educational_Summary_Report_10934.pdf]

MedEdPORTAL® The AAMC Journal of Teaching and Learning Resources

Original Publication Open Access

Pharmacology, Pharmacotherapy, and Pharmacopolicy Through an Evidence-Based Medicine: A Novel Approach for First-Year Medical Students

Alexander M. Mozeika, PharmD*, Rijul Asri, James F. Theis, PhD, Carolyn K. Suzuki, PhD

*Corresponding author: alexander.m.mozeika@gmail.com

Abstract

Introduction: As evidenced by student performance on various assessments, pharmacotherapy remains a comparative weakness in undergraduate medical education, with several institutions developing novel strategies for students to apply these principles in a practical setting. Medical curricula have recently prioritized group-learning modalities and evidence-based medicine education. However, these principles have yet to impact pharmacology education. We developed and implemented an evidence-based, group-learning exercise for first-year medical students focusing on pharmacology through the practical lens of pharmacotherapy and pharmacopolicy.
Methods: First-year medical students in different groups were assigned a particular medication and, during an in-class session, were encouraged to meet with other representatives assigned the same drug to interpret the provided package insert and any online information. Students then reconvened with their groups to engage in collaborative teaching about each assigned drug before completing a group quiz using online resources. Facilitators reviewed the group quiz and allowed time for student questions. **Results:** For 180 participants, the average group-quiz score was 86%, ranging from 68% to 100%. Student-reported satisfaction with the activity in meeting its preset objectives averaged 3.7 on a 5-point scale, with 5 being most positive. **Discussion:** Overall, this activity effectively integrates principles of pharmacotherapy and pharmacopolicy into a group-based, evidence-based exercise. Limitations of the activity include the number of possible example drugs and the amount of material covered in a given time frame. However, the activity lends itself to the role of an introductory session in a longer curriculum centered on clinical-applied pharmacology and evidence-based practice.

Keywords
Pharmacology, Pharmacotherapy, Pharmaceutical Policy, Evidence-Based Medicine, Pharmacology and Toxicology

Educational Objectives

By the end of this activity, learners will be able to:

1. Apply the regulatory principles of the Food and Drug Administration (FDA) and United States Drug Association in the context of the evaluation of clinical trials and drug markets.
2. Compare and contrast FDA-approved prescription and generic drugs, over-the-counter drugs, and dietary supplements in the development of patient-specific care plans.
3. Apply knowledge of mechanisms of action, intended health outcomes, and potential adverse effects of the specific drugs and dietary supplements assigned to determine the best course of action for treatment and monitoring in clinical scenarios.
4. Integrate theoretical knowledge with a targeted, evidence-based consultation of drug databases in order to make real-time optimal treatment decisions for specific clinical scenarios.

Citation:
Mozeika AM, Asri R, Theis JF, Suzuki CK. Pharmacology, pharmacotherapy, and pharmacopolicy through an evidence-based medicine: a novel approach for first-year medical students. *MedEdPORTAL*. 2020;16:10934.
https://doi.org/10.15766/mep_2374-8265.10934

Introduction

Over the past decade, the approach to preclinical medical education has evolved to incorporate novel teaching modalities, with marked success across several disciplines.[1-3] However, pharmacology and pharmacotherapy education remain areas of comparative weakness, as evidenced by student performance on various assessments.[4,5] Pharmacology curricula continue to utilize a heavily didactic component,[6] which proves less effective than the more-interactive learning styles used for other disciplines and may explain a part of this shortcoming.[7] Additionally, pharmacology is still taught with a

Copyright © 2020 Mozeika et al. This is an open-access publication distributed under the terms of the Creative Commons Attribution license.

focus on basic science principles. While these concepts are important, preclinical students generally respond better to the contextualization of these disciplines in clinical medicine.[8-10] This clinical context, as well as the translation of pharmacology into pharmacotherapy, relies heavily on principles of evidence-based medicine and pharmacopolicy, both of which influence delivery of care and speak to other weaknesses of the average medical student.[11-14] Thus, early exposure to these ideas will not only better prepare clinical students but may also augment the appreciation of pharmacology at the attitudinal level. Overall, a level of proficiency in these areas—pharmacotherapy, evidence-based decision-making, and pharmacopolicy—is expected in a medical student during the clinical years and contributes to a global appreciation for pharmacology. Thus, a potentially novel group exercise addressing and integrating these topics is relevant.

The target audience for this activity is the preclinical undergraduate medical student, ideally early in the curricula. Our audience was first-year medical students participating in a foundational basic science course. The target knowledge, skills, and attitudinal gaps for this activity are as outlined above, namely, pharmacology, pharmacotherapy, evidence-based medicine, pharmacopolicy, and drug regulations.

Current literature specifically published in *MedEdPORTAL* addresses the topics of group learning, pharmacology, pharmacotherapy, and evidence-based medicine through discrete exercises and activities.[15-18] Several studies published elsewhere validate a group methodology to develop and assess evidence-based medicine skills.[10-12] However, these exercises focus on diagnostics and nonpharmacological management, whereas our activity employs this approach specifically for pharmacotherapy. Conversely, the diabetes mellitus program from Goedde, Tracy, D'Epiro, and Gilbert[15] approaches pharmacotherapy education through group learning but lacks the evidence-based lens herein discussed. The value of consulting literature when making treatment decisions, especially for complicated patients, emerges through previously generated workshops,[14] but these activities focus more on a single resource without systematically guiding students through the process of reconciling multiple resources. Our activity combines each of these styles and areas of education by encouraging preclinical students in an undergraduate medical education program to utilize evidence-based strategies in their evaluation of pharmacotherapeutic decisions, using the model of group learning. Furthermore, our activity contextualizes this within food and drug regulation and policy, a topic that is notably deficient in the current scholarly milieu but that plays a role in practical clinical decision-making. Thus, our module attempts to utilize an interactive and integrative exercise to approach pharmacology in the context of evidence-based medicine and pharmacopolicy, which constitutes a novel contribution to *MedEdPORTAL*.

Methods

We implemented this activity as the third of a four-session series during the first foundational curricular block of the medical curriculum, focusing on molecular biology, genetics, biostatistics, pharmacology, and cell physiology. These four sessions served to illustrate clinical connections for the basic science aspects of the course. We administered the educational series weekly over the course of 4 weeks, and the topics of each session in order were genetics of sickle cell anemia, glycogen storage diseases, evidence-based pharmacotherapy, and nutrition. Facilitators required a working knowledge base of Food and Drug Administration (FDA) procedures and guidelines, of evidence-based medicine practices and resources, of basic pharmacologic and pharmacotherapeutic principles, and of the conditions and therapies presented in the cases, specifically, hypertension, hyperlipidemia, and pain management. Learners required a basic knowledge of pharmacologic and pharmacotherapeutic principles and of the conditions presented in the cases, specifically, hypertension, hyperlipidemia, and pain management.

We first met to generate the materials for the session, including the activity information sheet with simple background definitions (Appendix A) that detailed the structure of the session; the abridged United States Drug Association (USDA) drug-approval process informational sheet (Appendix B), adapted from content available on the publicly accessible USDA website[19]; the abridged FDA drug-approval process informational sheet (Appendix C), adapted from content on the publicly accessible FDA website[20]; the adverse event versus side effect informational sheet (Appendix D); a seating chart for the group assignments in our lecture hall (Appendix E); and the drug-specific handouts (Appendices F-L), adapted from package inserts for each of the medications. We also generated the group quiz (Appendix M) and the answer key for the group quiz (Appendix N). We generated the knowledge test by referencing the learning objectives and creating a series of questions that we believed addressed each one; we then met to review the questions generated and selected the final group in order to minimize repetition of concepts.

We randomly assigned six students to one main small group. We did not take any student-specific factors into consideration; we

Copyright © 2020 Mozeika et al. This is an open-access publication distributed under the terms of the Creative Commons Attribution license.

did not let students self-form groups. We then assigned each student within the main small group to a specific color, effectively creating color groups. We assigned each group color a specific drug handout to review prior to and during the session.

We posted group assignments and the advance preparation resources to our learning management system for student access a week before the session. Additionally, we encouraged students to familiarize themselves with Lexicomp Online,[21] the goal being for them to use this database as an optional resource during the activity and assessment. During the session, we distributed hard copies of all of the preparation resources and split students into color groups according to the seating chart we generated. After 30 minutes of discussion, we encouraged the color-coded groups to assemble and discuss the drug-specific handouts. After another 30 minutes of discussion, we distributed the group quiz. Groups were given 25 minutes to complete the group quiz, which contained 19 multiple-choice questions pertaining to the session topics, including the drug-regulation process, specific facts about the drugs presented during the session, and accessing databases for clinical decision-making. We allowed the groups to use any resources provided during the session, specifically encouraging the use of the drug-specific handouts and Lexicomp Online as an optional resource. Additionally, we did not limit access to any other resources or internet services. Upon completion of the group quiz, we reviewed the answers in a lecture-style format and allowed groups to ask content-related questions. At the close of the session, we allowed individual students to discuss questions and problems with us and determined our response on an individual basis. If the student cited evidence-based guidelines to appeal a question, we reviewed the clarity of the question and, if appropriate, revised the answer key accordingly. After the session, members of the office of education graded the exercise by hand and reported a final score out of 100% to students.

Facilitation Schema (2-hour time allotment):

1. Introduction to session/group breakdown: 10 minutes.
2. Drug-specific discussion (color groups): 30 minutes.
3. Summary discussion (small groups): 30 minutes.
4. Group quiz: 25 minutes.
5. Quiz review, questions, and feedback: 25 minutes.

Assessment

We assessed the impact of the session across each of the four objectives in Appendix A using the group quiz, a multiple-choice assessment that included questions on pharmacopolicy (objectives 1 and 2), fact-recall questions on specific pharmacotherapeutic agents (objective 3), and clinical application questions requiring use of drug databases (objective 4). Specifically, quiz questions 1, 2, 3, and 5 assessed objective 1; quiz questions 4 and 6 assessed objective 2; quiz questions 7, 8, 9, 10, 11, 12, 13, 14, and 15 assessed objective 3; and quiz questions 16, 17, 18, and 19 assessed objective 4.

We assessed student-reported feedback through a comprehensive end-of-course survey that included three statements specific to this particular activity, not to the other activities in the series, rated on a 5-point scale.

Results

First-year medical students enrolled in the Foundations of Medicine course at Rutgers New Jersey Medical School during the fall semester of 2017 ($N = 180$) participated in the activity.

We assessed student performance on the group quiz as a percentage of questions answered correctly. Of 180 participants, the lowest reported score was 68% (13 questions correct), and the highest reported score was 100% (19 questions correct). The mean score was 86%, the median score was 84%, and the mode score was 90%.

Feedback questions were rated on a 5-point scale (1 = *hardly at all*, 2 = *to a small degree*, 3 = *to a moderate degree*, 4 = *to a considerable degree*, 5 = *to a very high degree*). For feedback question 1, “Worthwhile information was obtained from [the exercise] about FDA oversight, prescription drugs, OTC [over-the-counter] drugs and supplements, associated precautions,” the average student response was 3.81. For feedback question 2, “Learning about Lexicomp from [the exercise] will be a useful resource in the future,” the average student response was 3.67. For feedback question 3, “The format of [the exercise] which involved individual learning and group teaching and learning was productive,” the average student response was 3.63.

Discussion

To address the potential shortcomings in pharmacology curricula at the level of the preclinical undergraduate medical student, we developed an interactive group exercise that contextualizes these concepts in the principles of evidence-based medicine and pharmacopolicy, both of which are necessary for clinical decision-making.

Overall, this exercise addresses several otherwise-lacking components of pharmacotherapy education in a cohesive way by integrating principles of pharmacology, pharmacotherapy,

Copyright © 2020 Mozeika et al. This is an open-access publication distributed under the terms of the Creative Commons Attribution license.

pharmacopolicy, and evidence-based medicine. The structure of the exercise involves group-learning with open access to resources, which limits the amount of baseline medical knowledge required prior to the activity and thus lends itself well to the early preclinical undergraduate medical student. Our implementation of the activity was generally well received, with specific student feedback and concerns outlined in the sections below. Students were able to meet each of the aims, as defined through the individual components of the group quiz.

We developed this exercise through a partnership between educators and students, with the latter group being able to draw from their recent experiences to inform the structure of the session and to address real-time gaps in the curriculum. We found the reliance on student input to be the most-significant aspect of this exercise as well as the most generalizable to other curricular innovations. We incorporated this activity into a preexisting series of small-group activities, which facilitated its implementation and integration into the curriculum. Based on student responses to end-of-exercise surveys, student reception of the activity was also positive because it provided a clinical and policy perspective on topics from that specific point in the course. Our group quiz, as constructed, appropriately addressed the objectives set forth through discrete questions, and while the quiz could be expanded, we maintain that it was an appropriate assessment for the time allocated. Student performance on the group quiz reflected the development of competency across all objectives.

One potential limitation to this resource arises from the collection of agents presented to students as appendices. Our exercise provides students with examples of common pharmacologic agents with a broad spectra of activity that respond to relatively common pathologies, allowing students to approach the exercise without a significant amount of formalized medical training. However, because these agents are common, we cannot control for each student's baseline level of knowledge about the drug, which complicates the analysis of exactly how much this exercise augments their understanding of those therapeutics. Another limitation is the length of the group quiz vis-à-vis the time allocated for the session. Although the current resource addresses each objective, a longer exercise period may allow for a more-thorough assessment in each category. Finally, the nature of the exercise in requiring students to consult outside resources provides a challenge in grading if a student finds conflicting information from reputable resources. While this is not common, facilitators must be prepared to exercise some discretion when students present peer-reviewed articles regarding a specific answer choice.

This activity introduces several key principles to students, including pharmacology, pharmacotherapy, pharmacopolicy, and evidence-based medicine, through one discrete exercise. Future projects will focus on providing more education on each of these topics, especially through clinical case scenarios. Specifically, to reinforce the practical nature of this exercise, we developed an integrative curriculum that builds upon students' utilization of drug databases by tasking them to approach treatment decisions for complicated patient case scenarios. We also hope to expand the exercise into a more-thorough examination of health policy that even better acquaints students with the regulations within which they will practice, including pharmacoeconomics and pharmacovigilance, to improve student knowledge generally about health care policy.

Appendices

A. Activity Information.docx
B. USDA QuickSheet.pdf
C. FDA QuickSheet.pdf
D. Adverse vs Side Effects.docx
E. Seating Chart.pdf
F. Acetaminophen Handout.pdf
G. Beano Handout.docx
H. Mevacor Handout.pdf
I. Naproxen Handout.pdf
J. Praluent Handout.pdf
K. Xenical Handout.pdf
L. Fat-Soluble Vitamins Handout.pdf
M. Group Quiz.docx
N. Quiz Answers.docx

All appendices are peer reviewed as integral parts of the Original Publication.

Alexander M. Mozeika, PharmD: Fourth-Year Medical Student, Department of Education, Rutgers New Jersey Medical School; ORCID: https://orcid.org/0000-0003-4037-7526

Rijul Asri: Medical Student, Department of Education, Rutgers New Jersey Medical School

James F. Theis, PhD: Adjunct Assistant Professor, Department of Biochemistry & Molecular Biology, Rutgers New Jersey Medical School

Carolyn K. Suzuki, PhD: Associate Professor, Department of Biochemistry & Molecular Biology, Rutgers New Jersey Medical School

Copyright © 2020 Mozeika et al. This is an open-access publication distributed under the terms of the Creative Commons Attribution license.

Acknowledgments
We would like to acknowledge Dr. Carol Lutz for providing the lecture on prostaglandins.

Disclosures
None to report.

Funding/Support
None to report.

Ethical Approval
Reported as not applicable.

References

1. Thompson BM, Schneider VF, Haidet P, et al. Team-based learning at ten medical schools: two years later. *Med Educ*. 2007;41(3):250-257. https://doi.org/10.1111/j.1365-2929.2006.02684.x
2. Parmelee DX, DeStephen D, Borges NJ. Medical students' attitudes about team-based learning in a pre-clinical curriculum. *Med Educ Online*. 2009;14:4503. https://doi.org/10.3402/meo.v14i.4503
3. Candler C, Ihnat M, Huang G. Pharmacology education in undergraduate and graduate medical education in the United States. *Clin Pharmacol Ther*. 2007;82(2):134-137. https://doi.org/10.1038/sj.clpt.6100266
4. Engels F. Pharmacology education: reflections and challenges. *Eur J Pharmacol*. 2018;833:392-395. https://doi.org/10.1016/j.ejphar.2018.06.032
5. Richir MC, Tichelaar J, Geijteman ECT, de Vries TPGM. Teaching clinical pharmacology and therapeutics with an emphasis on the therapeutic reasoning of undergraduate medical students. *Eur J Clin Pharmacol*. 2008;64(2):217-224. https://doi.org/10.1007/s00228-007-0432-z
6. de Vries TPGM, Henning RH, Hogerzeil HV, et al. Impact of a short course in pharmacotherapy for undergraduate medical students: an international randomised controlled study. *Lancet*. 1995;346(8988):1454-1457. https://doi.org/10.1016/S0140-6736(95)92472-8
7. Knight JK, Wood WB. Teaching more by lecturing less. *Cell Biol Educ*. 2005;4(4):298-310. https://doi.org/10.1187/05-06-0082
8. Dahle LO, Brynhildsen J, Fallsberg MB, Rundquist I, Hammar M. Pros and cons of vertical integration between clinical medicine and basic science within a problem-based undergraduate medical curriculum: examples and experiences from Linköping, Sweden. *Med Teach*. 2002;24(3):280-285. https://doi.org/10.1080/01421590220134097
9. Duban S, Mennin S, Waterman R, et al. Teaching clinical skills to pre-clinical medical students: integration with basic science learning. *Med Educ*. 1982;16(4):183-187. https://doi.org/10.1111/j.1365-2923.1982.tb01245.x
10. Kulasegaram KM, Martimianakis MA, Mylopoulos M, Whitehead CR, Woods NN. Cognition before curriculum: rethinking the integration of basic science and clinical learning. *Acad Med*. 2013;88(10):1578-1585. https://doi.org/10.1097/ACM.0b013e3182a45def
11. Del Mar C, Glasziou P, Mayer D. Teaching evidence based medicine. *BMJ*. 2004;329:989. https://doi.org/10.1136/bmj.329.7473.989
12. Gonzalo JD, Dekhtyar M, Starr SR, et al. Health systems science curricula in undergraduate medical education: identifying and defining a potential curricular framework. *Acad Med*. 2017;92(1):123-131. https://doi.org/10.1097/ACM.0000000000001177
13. Gonzalo JD, Haidet P, Blatt B, Wolpaw DR. Exploring challenges in implementing a health systems science curriculum: a qualitative analysis of student perceptions. *Med Educ*. 2016;50(5):523-531. https://doi.org/10.1111/medu.12957
14. Straus SE, Glasziou P, Richardson WS, Haynes RB. *Evidence-Based Medicine: How to Practice and Teach EBM*. 5th ed. Elsevier; 2019.
15. Goedde M, Tracy P, D'Epiro G, Gilbert M. Pharmacotherapy of type 2 diabetes: team-based learning. *MedEdPORTAL*. 2016;12:10395. https://doi.org/10.15766/mep_2374-8265.10395
16. Goodell M, Musil B. A longitudinal pharmacotherapy curriculum in family medicine residency education. *MedEdPORTAL*. 2007;3:587. https://doi.org/10.15766/mep_2374-8265.587
17. Strano-Paul L, Lane S. Diagnosis and treatment of obesity: a TBL exercise for third-year medical students. *MedEdPORTAL*. 2015;11:10010. https://doi.org/10.15766/mep_2374-8265.10010
18. Triantafylidis LK, Phillips SC, Hawley CE, Schwartz AW. Finding the Sweet Spot: an interactive workshop on diabetes management in older adults. *MedEdPORTAL*. 2019;15:10845. https://doi.org/10.15766/mep_2374-8265.10845
19. Development & approval process: drugs. U.S. Food and Drug Administration. Updated October 28, 2019. https://www.fda.gov/drugs/development-approval-process-drugs
20. FDA drug approval process infographic (horizontal). U.S. Food and Drug Administration. Accessed August 30, 2017. https://www.fda.gov/drugs/drug-information-consumers/fda-drug-approval-process-infographic-horizontal
21. Lexicomp Online. Lexicomp. Accessed November 27, 2019. https://www.lexi.com

Received: July 5, 2019
Accepted: January 21, 2020
Published: July 20, 2020

Copyright © 2020 Mozeika et al. This is an open-access publication distributed under the terms of the Creative Commons Attribution license.
